# Modeling convergent scale-by-scale skin color patterning in multiple species of lizards

**DOI:** 10.1016/j.cub.2022.10.044

**Published:** 2022-12-05

**Authors:** Ebrahim Jahanbakhsh, Michel C. Milinkovitch

**Affiliations:** 1 Laboratory of Artificial & Natural Evolution (LANE), Department of Genetics & Evolution, University of Geneva, 1211 Geneva, Switzerland; 2 SIB Swiss Institute of Bioinformatics, 1211 Geneva, Switzerland

**Keywords:** cellular automaton, Lenz-Ising model, reaction-diffusion, Turing patterns, skin color patterns, lizards, scales, evolution, development, convergence

## Abstract

Skin color patterning in vertebrates emerges at the macroscale from microscopic cell-cell interactions among chromatophores. Taking advantage of the convergent scale-by-scale skin color patterning dynamics in five divergent species of lizards, we quantify the respective efficiencies of stochastic (Lenz-Ising and cellular automata, sCA) and deterministic reaction-diffusion (RD) models to predict individual patterns and their statistical attributes. First, we show that all models capture the underlying microscopic system well enough to predict, with similar efficiencies, neighborhood statistics of adult patterns. Second, we show that RD robustly generates, in all species, a substantial gain in scale-by-scale predictability of individual adult patterns without the need to parametrize the system down to its many cellular and molecular variables. Third, using 3D numerical simulations and Lyapunov spectrum analyses, we quantitatively demonstrate that, given the non-linearity of the dynamical system, uncertainties in color measurements at the juvenile stage and in skin geometry variation explain most, if not all, of the residual unpredictability of adult individual scale-by-scale patterns. We suggest that the efficiency of RD is due to its intrinsic ability to exploit mesoscopic information such as continuous scale colors and the relations among growth, scales geometries, and the pattern length scale. Our results indicate that convergent evolution of CA patterning dynamics, leading to dissimilar macroscopic patterns in different species, is facilitated by their spontaneous emergence under a large range of RD parameters, as long as a Turing instability occurs in a skin domain with periodic thickness.

**Video abstract:**

## Highlights

• Multiple lizard species convergently evolved dynamics of scale-by-scale color change
• Mesoscopic models predict pattern statistics without cellular and molecular variables
• Turing models generate robust predictions of individual scale-by-scale patterns
• Uncertainties in skin color and geometry explain patterns’ residual unpredictability

## Teaser

Jahanbakhsh and Milinkovitch analyze convergent scale-by-scale skin color patterning dynamics in five divergent species of lizards and show that reaction-diffusion models robustly yield scale-by-scale predictability of individual adult patterns without the need to parametrize the system down to its many cellular and molecular variables.

## Introduction

Skin color patterning in vertebrates, as extensively studied in zebrafish,^1^^,^^2^^,^^3^^,^^4^^,^^5^^,^^6^^,^^7^ is a self-organized process, i.e., the spatial segregation of chromatophores observed during development does not follow pre-patterned positional information but autonomously emerges from cell-cell interactions among the chromatophores themselves.^1^^,^^8^ Because some of these interactions occur at short ranges and others at long ranges,^1^^,^^6^ the process can be efficiently described in the reaction-diffusion (RD) mathematical framework.^1^^,^^8^^,^^9^^,^^10^^,^^11^^,^^12^^,^^13^^,^^14^ In addition, we have demonstrated^12^^,^^14^ that, in the ocellated lizard (*Timon lepidus*), reduction of skin thickness at the borders of skin scales robustly transforms^12^^,^^14^ the RD color patterning process into a stochastic cellular automaton (sCA) where the neighborhood of monochromatic (either green or black) skin scales defines their color-flipping probabilities. Furthermore, we have shown^15^ that the 16-parameter sCA model (8 parameters for the color-flipping probabilities of green scales with 0–7 green neighbors, and 8 equivalent parameters for black scales) can be mapped to a simpler 2-parameter Lenz-Ising model^16^^,^^17^ (developed in the 1920s for describing the behavior of ferromagnetic materials), which produces the observed steady-state neighborhood distribution of ocellated lizard adult patterns.

However, it remains unclear if the deterministic and continuous-state RD framework allows capturing features of the scale-by-scale skin color patterning process beyond those efficiently described with stochastic and discrete-state sCA and Lenz-Ising models. First, using a deterministic RD model might seem counter-intuitive because reactions and diffusion can be argued to be stochastic processes at the microscopic scale. However, RD is a continuous model that describes how components’ concentrations at the mesoscopic/macroscopic scales, i.e., averages of molecule/cell densities at the nanoscopic/microscopic scales, deterministically vary in time and space. This is similar to deterministic thermodynamical properties emerging at the macroscale from statistical mechanical properties of microscopic entities. Hence, if initial components’ concentrations are identical, RD simulations will always produce the same single mesoscopic trajectory. Second, as the RD,^1^^,^^8^^,^^9^^,^^10^^,^^11^^,^^12^^,^^14^ sCA, and Lenz/Ising models used to describe skin color patterning all equally ignore the unidentified or unmeasured underlying molecular and cellular variables (generally ill-defined as “noise”), it can be argued that the former has no a priori reason to better capture, than the two latter, the dynamics of the system. Still, we reason here that RD might provide an intrinsically better description of the scale-by-scale skin color patterning process because it implicitly integrates a relation between the pattern length scale and geometrical parameters (such as scale geometries and their growth), and it allows to exploit the continuous-state distribution of scale colors (especially important at initial condition). To test this conjecture, we investigate here if deterministic RD allows describing individual patterning trajectories beyond their statistical properties, i.e., can RD efficiently predict, in multiple species, the positions of black and green/yellow scales in individual adult lizards despite that underlying unknown cellular and molecular variables are ignored?

We consider RD models that involve time, state, and spatial discretization at different levels: (1) a discrete RD model in two dimensions (2D-dRD), which is discrete in space but continuous in state and time, formally derived from Turing’s spatially continuous RD equations by renormalizing the diffusion term;^12^ (2) a 2D continuous RD model (2D-cRD), which is continuous in space, state, and time; and (3) a bona fide tridimensional cRD model (3D-cRD^14^). We have shown^12^^,^^14^^,^^15^ that all these RD models compute dynamics that are either constitutionally (dRD) or effectively (2D cRD and 3D cRD) discretized in space (i.e., scales are essentially monochromatic at any time point) and near discretized in state and time (i.e., color switching, between green and black, is not instantaneous but is much faster than the overall patterning process). These discretizations make the dynamics particularly suitable to unambiguous quantitative investigation: as the number of scales and the topology of the lattice are invariants throughout the life of a lizard, one can unequivocally identify the positions of black scales and green/yellow scales at any given time point during the post-hatching development of the animal.

**Figure 1:**
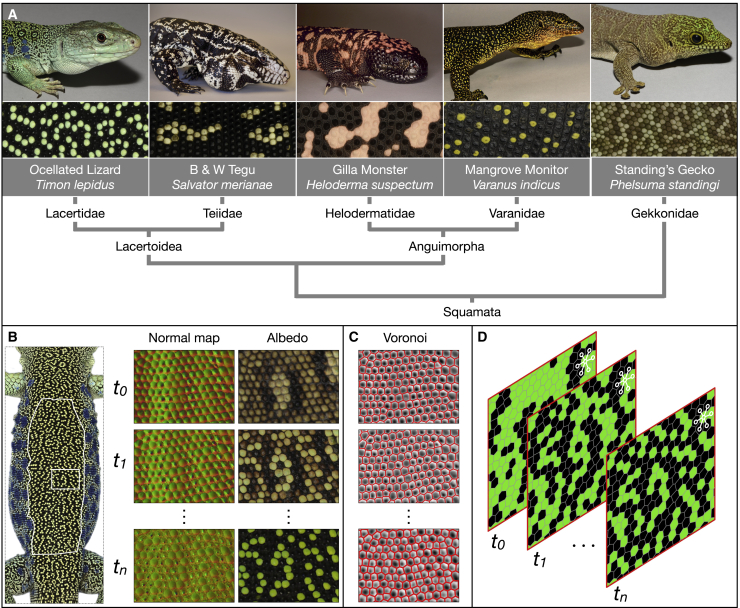
Acquisition of skin-scale color texture in five lizard species (A) The five species investigated here (top, image of an adult individual; middle, close up on the adult pattern) belong to divergent squamate lineages (bottom, phylogenetic cladogram). (B) A large dorsal skin patch (white outline in left panel) is photographed from different orientations and under different directions of incident illumination to generate high-resolution microgeometry (normal map, central panels, is shown for the small rectangular region framed in the left panel) and color texture (albedo, right panels) at multiple time points $t_{i}$. The individual shown in the left panel is TL1. (C) The filtered curvature field (shading) allows identifying scale centers that are then used for building a Voronoi diagram, identifying scale boundaries (red lines), and scale lattice connectivity. (D) Matching of scales across time points produces a space-time network of scales; white lines show one scale with one of its six neighbors shifting color from green to black between time points $t_{1}$ and $t_{n}$. See also Figure S6.

Here, we investigate whether these various stochastic and RD models of patterning can be generalized to five species, belonging to five divergent lineages, exhibiting largely different adult patterns (Figure 1A), but that all convergently evolved dynamics of post-hatching scale-by-scale color change: the ocellated lizard (*T. lepidus*), the Argentine black and white tegu (*Salvator merianae*), the Gila monster (*Heloderma suspectum*), the mangrove monitor (*Varanus indicus*), and the Standing’s day gecko (*Phelsuma standingi*). First, we show that all models predict neighborhood statistics of adult patterns with similar efficiencies. Second, we show that RD models substantially improve predictability of actual patterns beyond their statistical features without the need to parametrize the system down to its many cellular and molecular variables.^18^ More specifically, we show, in all five species, that continuous-state color information (i.e., estimates of RD-component concentrations based on the observed “greenness”/“blackness” of scales) from real juvenile lizards provides a substantial gain in scale-by-scale predictability of their corresponding adult patterns by RD over the stochastic Lenz-Ising and CA models. Third, we show that a substantial proportion of the residual unpredictability is due to heterogeneity in the variation of skin thickness among scale borders and among scales. Fourth, using Lyapunov spectrum analyses, we show that the uncertainty in color measurement in our experimental setup is sufficient to explain most, if not all, of the remaining residual unpredictability due to the non-linear dynamical nature of the system.

## Results

### Acquisition of lizard skin-scale color texture

Using a robotic system^19^ implementing the photometric stereo (PS) approach,^20^ the surface microgeometry (normal map) and color texture (RGB color albedo) of a large dorsal skin patch (Figure 1B) were acquired fourteen to forty-five times over a period of 2–4 years (starting from the juvenile stage) in individuals of five divergent species of lizards (Figure 1A): two ocellated lizards (TL1 and TL2, about 2,000 scanned scales each), two Argentine tegus (SM1 and SM2, about 1,600 scales each), four Gila monsters (HS1, HS2, HS3, and HS4, about 900 scales each), two mangrove monitors (VI1 and VI2, about 1,400 scales each), and two Standing’s day geckos (PS1 and PS2, about 3,000 scales each). Scale boundaries were identified as the edges of the Voronoi diagram (Figure 1C) partitioned using the scale centers defined by the watershed algorithm applied on the filtered curvature field derived from the initial normal map. This procedure both identifies scales and determines their neighborhood connectivity. Matching of scales across time points was performed by using local affine transformations producing a space-time network of scales (Figure 1D) with associated colors (albedo was averaged among pixels within each corresponding skin scale). The space-time network was used for finding optimal parameters in all stochastic and RD models. Additional details on animals, image acquisition, data processing, scale detection, scale matching, and iterative addition model parameter optimization are provided in the STAR Methods and in Tables S1 and S2.

### Prediction of neighborhood statistics

#### Stochastic cellular automaton

Our analyses of time series of ocellated lizard scale-color switching previously indicated^12^ that these dynamics can be effectively described as a stochastic cellular automaton (sCA), i.e., the stochastic automaton dynamically computes the adult labyrinthine pattern.^12^ Indeed, the probability of color change of a green/black scale at each time-discrete iteration of the sCA is a function of *n*_*g*__/__*b*_, i.e., the number of green/black direct neighbors. In real lizards, any black or green scale can have 0–7 isochromatic neighbors, such that the sCA model can be defined with 2 × 8 = 16 parameters. Here, we refine this model by using Bayesian inference to derive E[p], the expectation of the sCA probabilities (STAR Methods; Figure S1A), which are then used as CA probabilistic “rules” to simulate pattern time evolution. Each simulation is iterated until the number of scale-color flips reaches about the total number of flips observed in the real animal (between juvenile and adult stages); note that a perfect correspondence is usually not observed because one iteration of the sCA involves the color switch of more than one scale.

We then formulate an objective measure of how different the neighborhood statistics of each simulated pattern are from those of a real adult lizard. To this end, we simply calculate the normalized amplitude ($E_{16D}$) of the combined differences of simulated versus observed numbers of black and green scales with each of the 16 possible configurations of isochromatic neighbors. More technically, we compute, for each simulated adult pattern, the $L^{2}$ -norm of the 16-dimensional difference error vector ($E_{16D}$) of nearest-neighbor configuration statistics in comparison with the single observed adult pattern:

$$E_{16D}=\frac{1}{n_{s}}\sqrt{\sum_{i=0}^{7}{\left(n^{\mathrm{sim}}\left(\mathrm{Green},i\right)-n^{\mathrm{obs}}\left(\mathrm{Green},i\right)\right)}^{2}+{\left(n^{\mathrm{sim}}\left(\mathrm{Black},i\right)-n^{\mathrm{obs}}\left(\mathrm{Black},i\right)\right)}^{2}}$$

where $n_{s}$ is the number of scales whereas $n\left(\mathrm{Green},i\right)$ and $n\left(\mathrm{Black},i\right)$ are, respectively, the number of green and black scales with i isochromatic neighbors; superscripts ^sim^ and ^obs^ denote the simulation and observation, respectively.

**Figure 2:**
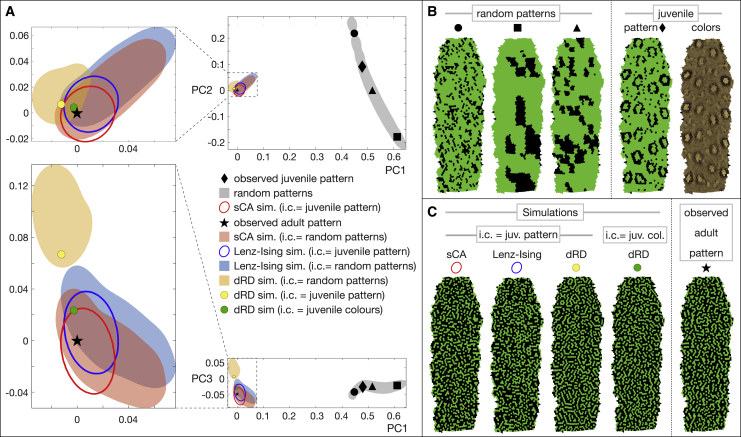
Prediction of neighborhood statistics in ocellated lizard individual TL1 (A) Projections on the principal component planes PC1-PC2 (top) and PC1-PC3 (bottom) of the 16D nearest-neighbor error vectors (in comparison with the observed adult pattern, black star) of patterns simulated with sCA (red ellipse and red shading), Lenz-Ising (blue), and dRD (yellow) models; interactive 3D graphs in PC1-PC2-PC3 space available as Data S1. Red ellipse, blue ellipse, and yellow spot show adult patterns simulated from the observed juvenile pattern (black diamond), whereas red, blue, and yellow shadings show adult patterns simulated from random patterns (gray area) as initial condition. The green spot shows the adult dRD-pattern simulated from the juvenile colors shown in (B) (rightmost panel). Ellipses and border of shadings indicate 1% density isolines. (B) Different initial conditions used for simulations; their localization in PC1-PC2-PC3 space is shown with the corresponding geometrical symbols. (C) Adult patterns simulated with different initial conditions (i.c.): juvenile (juv.) pattern (=scale colors thresholded to green or black) and juvenile colors (col.) are both shown in (B). Similar results are obtained for individual TL2 (Figure S2; Data S2). See also Figure S1 and Table S4.

We then use principal component analysis (PCA; Figure 2A) to identify the 3 largest modes (PC1, PC2, and PC3, corresponding to the 3 eigenvectors with largest eigenvalues) that are jointly capturing >90% of the variance of all error vectors. Performing 5,000 simulations, each starting from the real observed juvenile pattern (black diamond in Figures 2A and 2B), indicates that the sCA model is robust: multiple runs starting from this same initial condition evolve into a population of simulated adult patterns (one example is shown in Figure 2C) whose $E_{16D}$ values are restricted to an ellipsoid (red ellipses in Figure 2A; mean $E_{16D}$ ± SD = 0.037 ± 0.012) that also contains the zero-error coordinate (0,0,0) of the PC1-PC2-PC3 space, i.e., the position of the observed adult pattern (black star in Figures 2A and 2C). We then produce 5,000 random patterns that span all length scales (from one skin scale to about one-fourth of the length of the analyzed dorsal skin patch) and are characterized by a large range of initial $E_{16D}$ values (gray area in Figure 2A). Remarkably, the 5,000 simulations initiated from these random patterns (one simulation for each random pattern) show that the very large spread of initial conditions evolves into a population of patterns that are restricted to an area (red shading in Figure 2A; mean $E_{16D}$ ± SD = 0.065 ± 0.05) slightly larger than, but that strongly overlaps with, the patterns (red ellipses) simulated from the juvenile pattern. Very similar results are obtained for the second ocellated lizard individual analyzed here (TL2; Figure S2).

#### Lenz-Ising model

The Lenz-Ising model^16^^,^^17^^,^^21^ is a statistical mechanical model developed for describing the states of magnetic materials as a lattice of sites, each with one of two possible orientations (+1 or −1) of electronic/nucleic magnetic dipole moments. The model requires only two parameters: J, which defines how much neighbors with the same orientation are favored (J>0, ferromagnetic materials) or disfavored (J<0, antiferromagnetic materials), and B, which correspond to an “external” magnetic field favoring one or the other state (depending on the sign of B). We have recently shown^15^ that the time evolution of the Lenz-Ising model in thermal equilibrium effectively describes the dynamics of scale-by-scale skin color patterning of the ocellated lizard when we treat green and black scales as if they were +1 and −1 dipoles, respectively. More technically, the late-time probability distribution of patterns is equivalent to the canonical probability distribution of the antiferromagnetic Ising model at finite temperature ($1/k_{B}T>0$) with black scales being favored.^15^ Hence, the Lenz-Ising model is the simplest formalism (2 parameters instead of the sCA 16 parameters) describing the skin color pattern self-organization in the ocellated lizard.^15^

Similar to the sCA analyses above, we perform simulations using the Lenz-Ising model with βB and βJ parameters and dynamics,^15^ optimized for each individual (Figures S1B and S2B). The 5,000 Lenz-Ising simulations starting from the real juvenile pattern (black diamond) and the 5,000 simulations starting each from one of the random patterns (gray area) generate populations of $E_{16D}$ nearest-neighbor errors (Figure 2A, blue ellipses with mean $E_{16D}$ ± SD = 0.048 ± 0.016 and blue area with mean $E_{16D}$ ± SD = 0.091 ± 0.058, respectively) very similar to those obtained with the sCA model (red ellipses and red shading). These results indicate that, for ocellated lizards, the sCA and Lenz-Ising models exhibit similar performances in robustly predicting the nearest-neighbor statistics, hence the general labyrinthine look, of real adult patterns (Figure 2C).

#### Optimized reaction-diffusion models

RD models, first introduced by Turing,^9^ compute the concentrations of diffusing and reacting molecular species (RD components) through time and space. In the framework of skin color patterning, “diffusion” should be understood as “effective diffusion”^22^ of signals originating from short- and long-range cell-cell contacts.^1^ Note that the long-range interactions involve macrophage-like cells^6^ that recognize and drag plasma membrane blebs on xanthoblasts, generating and extending thin cellular projections (airinemes), before performing a random walk and ultimately depositing the airineme vesicle on the surface of a melanophore at a distance up to several xanthoblast-body lengths. It is likely that these cell-cell interactions have the capacity of generating long-ranged gradients (i.e., larger than the typical 1 mm skin scale of an adult ocellated lizard).

Here, we use a three-component (u,v,w) 2D continuous RD (cRD) model, initially developed to study skin color patterning in zebrafish.^1^ The components u and v represent the densities of melanophores and xanthophores (and their corresponding short-range factors), respectively, whereas w represents a long-range factor with diffusion coefficient $D_{w}$ much larger than $D_{u}=D_{v}$. We previously adapted this model for describing skin-scale color change dynamics in the ocellated lizard^12^ and extended it to 3D^14^ to take into account variation of the domain thickness. Here, we develop a new implementation of the 3D-cRD model in which skin geometry is represented as a curvilinear 3D grid (more accurate than the rectilinear grid used in Fofonjka and Milinkovitch^14^) yielding lower errors in computing components’ concentrations, hence allowing for the use of more efficient coarse-grain grids (STAR Methods).

Simpler modeling of the scaled skin color patterning process in 2D (i.e., in the plane of the skin) rather than in 3D can be achieved^12^ by taking into account the reduction of skin thickness at scale borders through scaling of the continuous RD diffusion coefficients (by a factor P) at the one-dimensional edges of the 2D scales. Beside this 2D continuous model (2D-cRD), we also introduced a discrete RD model (2D-dRD) where each scale is represented by a single node.^12^ This discretization is justified by the 3D geometry of the skin: as scale skin is much thicker than interscale skin, the concentrations of morphogens within a given skin scale tend to spatially homogenize, hence, each of these concentrations can be represented by a single value (i.e., the average concentration within the corresponding skin scale). This assumption is validated by bona fide 3D-RD simulations^14^ indicating that scales tend to effectively change color as a whole entity, rather than exhibit sustained gradients. We have previously shown how to renormalize the diffusion term when deriving the 2D-cRD model for regular hexagonal lattices.^12^ Here, we generalize the 2D-dRD model by deriving the discrete RD equations for arbitrary polygons in order to simulate the ocellated lizard skin color change dynamics on realistic (non-strictly hexagonal) lattices of skin scales. Details on the derivations of the 2D-cRD and 2D-dRD models are provided in the STAR Methods.

Furthermore, we suggest here improvements of the 2D-cRD and 2D-dRD models to increase their predictive power by (1) integrating growth of the domain in which patterning is taking place, (2) considering continuous variation of color states, and (3) optimizing RD parameters using Bayesian machine-learning global minimization based on a Gaussian process regression approach. To this end, we first assume that scales grow isotropically following a logistic function (parameters fitted using measurements from real lizards) that we then integrate in the RD equations. Second, using linear stability analysis close to the homogeneous steady state (HSS), we develop transformation functions to transfer juvenile scale CIELAB color information to the space of RD variables and use PCA to perform transformations, at later (post-juvenile) time points, from the u,v,w RD space to the CIELAB color space. Additional details on the growth-integrated RD models, transformation functions, linear stability analysis, PCA, and Bayesian optimization are given in the STAR Methods.

We then run 2D-dRD simulations from the same initial conditions as for the sCA and Lenz-Ising simulations. The 5,000 2D-dRD simulations starting each from one of the random patterns (gray area in Figure 2A) generate a population of patterns exhibiting error vectors (orange shading; mean $E_{16D}$ ± SD = 0.11 ± 0.029) similar to those obtained with the stochastic models (sCA and Lenz-Ising; red and blue shadings, respectively). When using the same single observed juvenile pattern (black diamond; Figure 2A) as that used for sCA and Lenz-Ising simulations (i.e., with scale colors thresholded to green or black by applying K-mean clustering), the resulting dRD-simulated adult pattern (yellow spot; Figure 2C) exhibits a nearest-neighbor error vector ($E_{16D}$ = 0.074; yellow spot; Figure 2A) situated in the same region of the PCA graph. However, one of the interests of RD is the possibility to exploit the information included in the continuous-state values of scales at the observed initial condition: the dRD simulation starting from these real colors of the juvenile pattern (top right image in Figure 2B), instead of a thresholded initial pattern with only two states, produces a pattern (green spot in Figures 2A and 2C; $E_{16D}$ = 0.033) that is closer to the observed adult pattern. Note that sCA and Lenz-Ising simulations cannot be performed with juvenile continuous color values as initial condition because these are intrinsically two-state models. Very similar results are obtained for the second ocellated lizard individual (TL2; Figure S2C).

#### Comparable neighborhood predictions of stochastic and RD models

The analyses above indicate that, for ocellated lizards, simulations initiated from the observed juvenile pattern, and run using any of the above models, generate simulated adult patterns with neighborhood statistics, hence, qualitative looks (Figures 2C and S2E), remarkably similar to those of the real adult pattern. In other words, although RD can exploit the information associated with the continuous color values observed in juveniles, it does not perform spectacularly better than sCA and Lenz-Ising two-state models for predicting the neighborhood statistics of adult patterns.

#### Prediction of neighborhood statistics in other species

**Figure 3:**
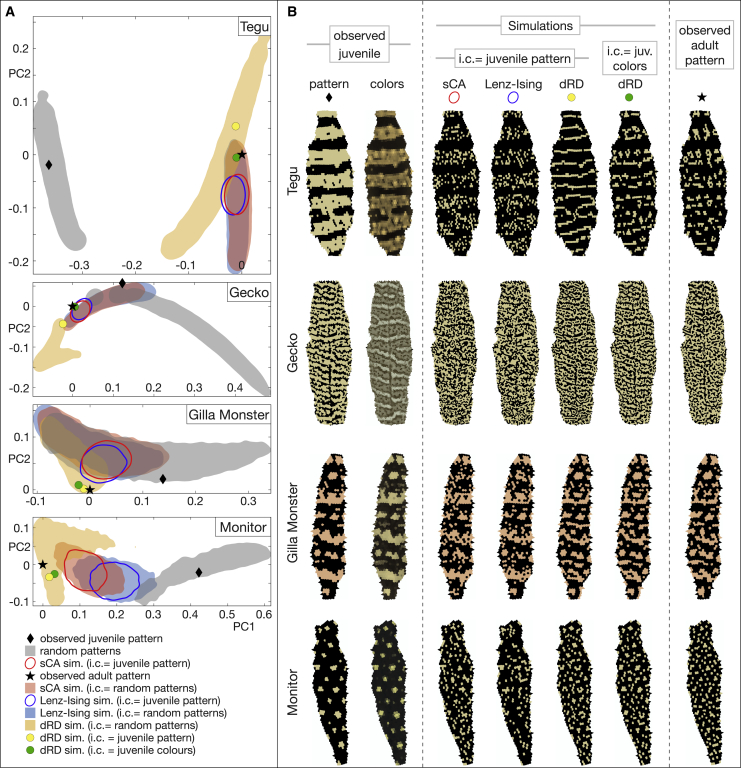
Prediction of neighborhood statistics in four additional species (A) Projections on the PC1-PC2 plane of the 16D nearest-neighbor error vectors (in comparison with observed adult pattern, black stars) of patterns simulated with sCA (red ellipse and shading), Lenz-Ising (blue), and dRD (yellow) models in black and white tegu (SM1), Standing’s gecko (PS1), Gila monster (HS1), and mangrove monitor (VI1). Red ellipses, blue ellipses, and yellow spots show adult patterns simulated from the observed juvenile pattern (black diamonds), whereas red, blue, and yellow shadings show patterns simulated from random patterns (gray areas) as initial condition. The green spots show the dRD-pattern simulated from the juvenile color shown in (B). (B) Left: observed juvenile patterns and colors. Center: adult patterns simulated with juvenile (juv.) pattern (=scale colors thresholded to green or black) or juvenile colors as initial condition (i.c.). Right: observed adult patterns. Ellipses and border of shadings indicate 1% density isolines. See also Tables S3 and S4 and Figure S3.

Our analyses indicate that both stochastic models (sCA and Lenz-Ising) are robust in all four additional species in predicting neighborhood statistics (Figure 3): performing 5,000 simulations, each starting from the real observed juvenile pattern (black diamond), generates a population of simulated adult patterns that are restricted to zones (Figure 3A, red and blue ellipses for sCA and Lenz-Ising, respectively) close to the zero-error coordinate (0,0) of the PC1-PC2 plane, i.e., close to the position of the observed adult pattern (black star). Note, however, that different species exhibit different lengths of $E_{16D}$ trajectory between juvenile and adult patterns because juveniles are closer to the HSS in some species (e.g., ocellated lizard) than in others (e.g., Gilla monster and mangrove monitor), such that they differ in their typical number of post-hatching scale-color flips. Regarding Lenz-Ising optimization, our analyses indicate that the model is antiferromagnetic for the gecko (as in the ocellated lizard), i.e., favoring labyrinthine patterns in which direct neighbors tend to exhibit opposite colors, and ferromagnetic for the tegu, i.e., favoring the existence of larger areas of isochromatic scales, whereas it is virtually on the boundary between ferromagnetic and antiferromagnetic in the Gila monster and mangrove monitor (Figure S3A).

As with the ocellated lizard (Figure 2), the dRD simulations initiated from either the thresholded yellow/orange versus black juvenile pattern or the real juvenile colors (Figure 3B, first and second column of images, respectively) produce adult patterns exhibiting a low nearest-neighbor error (yellow and green spots, respectively), indicating that RD is an excellent neighborhood configuration predictor in all species analyzed here, despite the substantially different motifs of their patterns. Also similar to the ocellated lizard, the error vectors generated in the four additional species with 2D-dRD simulations (orange areas in Figure 3A) starting from 5,000 random patterns are not particularly better than those obtained with the sCA and Lenz-Ising models (red and blue areas, respectively). Very similar results are obtained for the additional individuals analyzed here for the four species (SM2, PS2, HS2, HS3, HS4, and VI2; Figure S3B).

### Prediction of individual scale-by-scale patterns

We define for all models the “scale-by-scale error” between a simulated and observed pattern as

$$E_{\mathrm{sbs}}^{\left(k\right)}=\frac{1}{n_{s}}\sum_{i=1}^{n_{s}}\frac{\left\|C_{i}^{\mathrm{sim}}\left(\tau_{k}\right)-C_{i}^{\mathrm{obs}}\left(\tau_{k}\right)\right\|}{\left\|C_{b}\left(\tau_{k}\right)-C_{g}\left(\tau_{k}\right)\right\|}$$

where $n_{s}$ is the number of scales; $C_{i}$ denotes the color of the *i*^th^ scale; superscripts ^sim^ and ^obs^ denote the simulation and observation, respectively; $\tau_{k}$ denotes the time point; and $C_{g}$ and $C_{b}$ are the green and black state colors, respectively. For dRD analyses, $C_{i}$ are computed according to Equation 31 (STAR Methods), whereas $C_{g}$ and $C_{b}$ are mean values computed by K-mean clustering in which scale colors are defined in CIELAB coordinates. For sCA and Ising simulations, $C_{i}$ , $C_{g}$ , and $C_{b}$ are all replaced by the scalars representing the binary (“green” or “black”) state of scales.

#### Stochastic versus deterministic models

**Figure 4:**
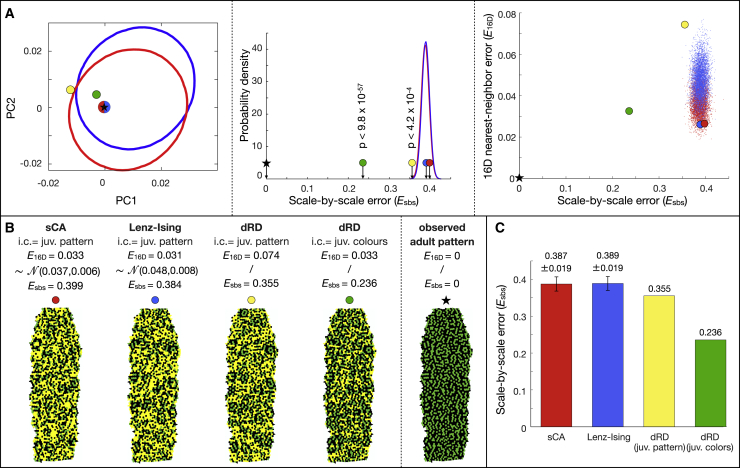
Prediction of scale-by-scale patterns in ocellated lizard individual TL1 (A) Simulated adult patterns with low neighborhood errors do not show particularly low scale-by-scale errors ($E_{\mathrm{sbs}}$) within the distribution of simulated patterns: red and blue spots indicate zero-error patterns (in the PC1-PC2 plane, left) among the 5,000 simulated with sCA and Lenz-Ising, respectively. Ellipses in left panel are 1% density isolines of neighborhood error vectors whereas the Gaussian distributions in the central panel are the $E_{\mathrm{sbs}}$ probability density functions. The adult patterns simulated from the juvenile pattern or juvenile colors (yellow and green spots, respectively) exhibit statistically smaller $E_{\mathrm{sbs}}$ (p values shown in central panel) than the sCA and Lenz-Ising probability density functions. Right: no correlation is observed between neighborhood error and scale-by-scale error; red points and blue points are 5,000 simulations with sCA and Lenz-Ising, respectively. (B) Simulated adult patterns (corresponding to red, blue, yellow, and green spots in A); scales whose color is erroneous (in comparison with observed adult, rightmost pattern) are indicated in yellow. (C) Histogram (mean ± SD) comparing scale-by-scale errors of adult patterns simulated with different models. Similar results are obtained for individual TL2 (Figure S2). See also Figure S5.

Stochastic models (sCA and Lenz-Ising) are susceptible to generating substantial errors in the prediction of individual scale-by-scale patterns despite the fact that they excel in predicting the bulk statistics inferred from them. Indeed, given enough iterations of the sCA process or long enough thermalization of the Lenz-Ising model, the exact positions of green and black scales on a simulated adult pattern can, in principle, greatly differ from those observed on a real pattern, even if the neighborhood distributions between simulated and observed patterns are highly similar. We confirm this prediction by showing that sCA-simulated or Lenz-Ising-simulated patterns selected for exhibiting very low nearest-neighbor error vectors in the PC1-PC2-PC3 space (red and blue dots in Figure 4A, left) do not show $E_{\mathrm{sbs}}$ values particularly smaller than those observed for simulated patterns with larger $E_{\mathrm{sbs}}$ errors (Figure 4A, ellipses in left panel and distributions in central panel). This result is confirmed by the absence of correlation between sCA- or Lenz-Ising-associated $E_{16D}$ nearest-neighbor errors and their corresponding $E_{\mathrm{sbs}}$ scale-by-scale errors (Figure 4A, right).

On the other hand, RD models could, in principle, exhibit increased precision in predicting actual scale-by-scale patterns (and not only the bulk statistics inferred from them) for three reasons: (1) RD is deterministic; (2) RD integrates an implicit relation between, on one hand, the pattern typical length scale, and on the other hand, growth and the geometry of skin scales (i.e., their sizes as well as their numbers and lengths of edges); and (3) RD can exploit the information provided by the actual (u,v,w) RD-component concentrations at initial condition. Note that the (u,v,w) values can be estimated from the observed “greenness”/“blackness” of scales in real juvenile lizards using linear stability analysis and transformation functions (STAR Methods). Figures 4A–4C indicate that this conjecture of substantial gain in scale-by-scale predictability of observed adult patterns by RD over stochastic models is correct: starting from the same juvenile K-mean thresholded (green and black) pattern as for the stochastic models, RD generates a simulated pattern (yellow spot in Figures 4A and 4B) with a scale-by-scale error ($E_{\mathrm{sbs}}=35.5\%$) smaller than those obtained with the stochastic models. Although this improvement is statistically significant (p < 4.2 × 10^−4^), its amplitude is somewhat small (Figure 4C). Conversely, the improvement of scale-by-scale predictability becomes much more prominent (>15%, p < 9.8 × 10^−57^; green spot in Figure 4A) when using the juvenile unthresholded colors as initial condition. This result is unanticipated as neighborhood statistics, and *not* scale-by-scale error, were used as the objective function during Bayesian optimization of RD parameters. The same error reduction of about 15% is observed between stochastic models and the full RD analysis for the second ocellated lizard analyzed here (TL2; Figure S2F).

#### Residual unpredictability and skin thickness spatial distribution

**Figure 5:**
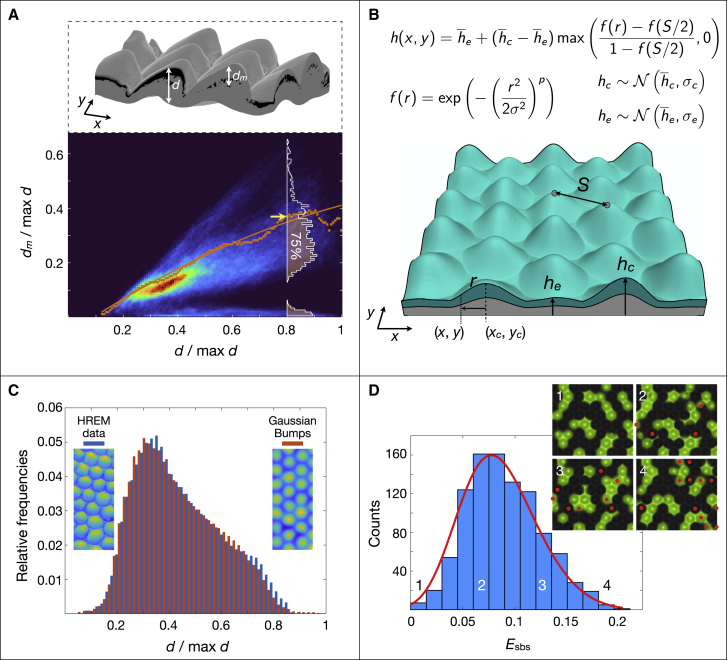
Heterogeneity in skin thickness variation affects RD predictability (A) Top: ocellated lizard dorsal 3D skin patch reconstructed using HREM; d and $d_{m}$ are, for each value of (x,y), the skin thickness and black pixel lowest depth, respectively. Bottom: heatmap of normalized $d_{m}$ versus normalized d; orange dots indicate, for each value of d/maxd, the boundary (yellow arrow) at the 75th percentile in the corresponding distribution (white profile); orange line, second-order polynomial smooth fit across the orange dots. (B) Example of an hexagonal 3D lattices of super-Gaussian bumps (mean distance among neighboring scales S is from HREM data; σ and p are Gaussian parameters in the equation on top) with $h_{c}$ (height for each bump) and $h_{e}$ (for each edge) are sampled from $N\left({\bar{h}}_{c},\sigma_{c}\right)$ and $N\left({\bar{h}}_{e},\sigma_{e}\right)$, where ${\bar{h}}_{c,e}$ and $\sigma_{c,e}$ are means and variances, respectively. The skin domain restricted to chromatophores (turquoise) is computed using the mapping $d_{m}\left(d\right)$; see text for details. (C) Histogram of domain thickness of the 3D lattice shown in (B) after optimizing σ, p, ${\bar{h}}_{c,e}$, and $\sigma_{c,e}$ to obtain a profile (red) highly similar to the HREM data histogram (blue). (D) Histogram of scale-by-scale error (in comparison with the pattern simulated with a reference homogeneous 3D domain of identical Gaussian bumps, i.e., $\sigma_{c}=\sigma_{e}=0$ ) in adult patterns simulated on 1,000 heterogeneous 3D domains similar to the one shown in (B). The fitted generalized extreme-value distribution (red line) indicates that heterogeneity in skin thickness variation produces a mean $E_{\mathrm{sbs}}\approx 8.5\%$. Inset shows examples of patterns generated with four heterogeneous domains (red dots indicate scales with wrong color, and numbers refer to their $E_{\mathrm{sbs}}$ value in the histogram).

Our experimental setup does not provide one key piece of information relevant to RD: the exact spatial distribution of skin thickness across the RD field. To quantify how much real skin geometry deviates from the assumption of identical skin thickness reduction at all scale borders, we perform here 3D geometry reconstruction of a patch of ocellated lizard dorsal skin using high-resolution episcopic microscopy (HREM^14^^,^^23^). Figure 5A shows a portion of the HREM-acquired skin 3D domain (black pixels correspond to melanophores) and the heatmap of black pixel positions ($d_{m}$) with respect to skin thickness (d). Whereas extraction of the spatial distribution of d is straightforward, the distribution of $d_{m}$ is obtained by mapping d to the second-order polynomial fit (orange line in Figure 5A) of 75% deepest observed black pixel positions for all scales. From these HREM data, we also extract the mean distance S among neighboring scales at their highest point, as well as the means among the top-surface highest point of scales (${\bar{h}}_{c}$) and among the heights of edges (${\bar{h}}_{e}$).

We then construct a 3D lattice of super-Gaussian bumps for which we optimize parameters σ and p (cf. equations in Figure 5B), as well as the standard deviations $\sigma_{c}$ and $\sigma_{e}$, of bump heights ($h_{c}$) and scale edge heights ($h_{e}$), respectively, to obtain a depth-map histogram (i.e., a skin thickness distribution) highly similar to that observed in the HREM data (Figure 5C). This lattice allows us to construct 1,000 noisy domains by sampling $h_{c}$ (for each bump) and $h_{e}$ (for each edge) from the normal distributions $N\left({\bar{h}}_{c},\sigma_{c}\right)$ and $N\left({\bar{h}}_{e},\sigma_{e}\right)$. Finally, using the mapping $d_{m}\left(d\right)$ discussed above, we compute the bottom boundary of each domain by subtracting $d_{m}$ from the top boundary, hence producing models of the skin domain restricted to chromatophores (turquoise volume in Figure 5B). As these 1,000 domains are all different, but they each exhibit thickness heterogeneities (among scale centers and among scale edges) similar to those of real lizards, we can use them as follows to test for their impact on the predictability of RD modeling. For each generated domain, we run a bona fide 3D-cRD numerical simulation (parameter values obtained from Bayesian optimization in TL1; see above) and compare the resulting adult pattern with that obtained with the simulation performed on a reference homogeneous 3D domain of 64 *identical* super-Gaussian bumps (i.e., with the centers of all bumps set to ${\bar{h}}_{c}$ and all the edges set to ${\bar{h}}_{e}$). Note that the 1,000 simulations, and the reference simulation, are all started from the colors of a patch of 64 scales observed in TL1 at the juvenile stage. Figure 5D shows the distribution of scale-by-scale error associated to the 1,000 heterogeneous domains: the mean of the expected scale-by-scale error exclusively due to heterogeneity in skin thickness variation is $E_{\mathrm{sbs}}=8.5\%$. In other words, given the incomplete information on the thickness variation among scales and among scale borders, a hypothetical numerical model with perfect intrinsic predictability would, on average, still not allow predicting exact individual patterns in ocellated lizards with scale-by-scale error <8.5%. Details on HREM data acquisition and generation of noisy super-Gaussian 3D lattices are provided in the STAR Methods.

#### Residual unpredictability and color measurement uncertainty

In order to generate Turing instabilities, the partial differential equations used in RD must be non-linear. In the model we use, the non-linearity resides in the piecewise form of the reaction terms (Equation 13). Non-linearity could make the dynamical system sensitive to initial conditions: given that the colors of the scales cannot be known with infinite precision, the corresponding initial uncertainty in measurement is likely to grow with time. However, given that it generates Turing instabilities, the system we study must also be far from the chaotic regime where non-linearity would make it irreducibly unpredictable. As the dRD model exhibits a minimum predictability error of 23.6% in the analyses above, and about 8.5% of it is due to uncertainty regarding the spatial variation of skin thickness, what is the cause of the other 15.1%? Below, we show that most, if not all, of this residual unpredictability is due to uncertainty in color measurements at initial condition.

One powerful approach to estimate the degree of sensitivity of a dynamical system is to use Lyapunov spectrum analysis (STAR Methods), i.e., computing how fast two trajectories diverge when they initially differ by an arbitrary small difference. To this aim, we generate 2,000 “noisy initial conditions” by adding random noise to the colors of all scales of the observed juvenile lizard and study their time evolution as 2,000 trajectories in phase space. We then (1) measure the time evolution of the Euclidean distance $\delta_{\tau }=\left\|S_{\tau }-S_{\tau }^{\mathrm{ref}}\right\|$ between the state of each perturbed system ($S_{\tau }$) and the state ($S_{\tau }^{\mathrm{ref}}$) of the reference (i.e., the latter is the trajectory starting from the observed unperturbed juvenile colors) and (2) use the Lyapunov exponent λ as an estimate of the rate of divergence between the two trajectories (Figure S4).

**Figure 6:**
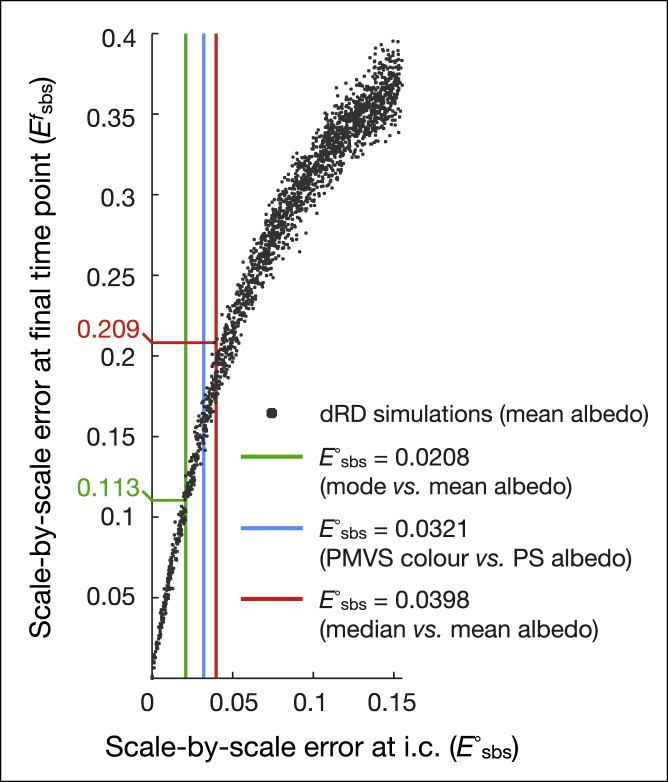
Scale-by-scale error due to juvenile color measurement uncertainty Plot of the scale-by-scale error at the final (adult) time point versus error at initial condition (i.c.) for the 2,000 simulations shown in Figure S4 (Lyapunov spectrum analysis). At initial (juvenile) condition, median albedo (red line) or mode albedo (green) or mean RGB colors (blue) give differences of scale colors (against mean albedo) that generate 11.3%–20.9% scale-by-scale error at the adult stage.

This analysis allows us to readily quantify the expected error at the adult stage given a specific uncertainty at the initial condition: we compute, for each of the 2,000 simulations, the scale-by-scale color errors, $E_{\mathrm{sbs}}^{\circ }$ and $E_{\mathrm{sbs}}^{f}$, i.e., at the juvenile and adult stages, respectively. Note that these errors are computed by comparing each simulation (starting with noisy initial condition) with the reference simulation (starting with the observed juvenile colors). Figure 6 shows the variation of $E_{\mathrm{sbs}}^{f}$ with respect to $E_{\mathrm{sbs}}^{\circ }$ across all 2,000 simulations: a larger initial error generates a larger error at the adult stage. To estimate the expected scale-by-scale error at the adult stage given the actual uncertainty in color measurements at the juvenile stage, we reflect that the color of each scale is measured as the mean albedo among pixels within that scale (STAR Methods). Although the procedure of using a single color for the whole scale is justified by the well-known behavior of the system^12^^,^^14^ (i.e., each of the (u,v,w) components tends to spatially homogenize within a scale), using the mean is arbitrary. Similarly, one could argue that using the mean RGB color observed on the pictures with homogeneous illumination is a more direct color measurement. Figure 6 indicates (vertical lines) that using the mode or the median (instead of the mean) of the PS albedo at initial condition would translate to an initial difference ($E_{\mathrm{sbs}}^{\circ }$) of 2.08% and 3.98%, respectively, whereas the use of RGB colors would correspond to an intermediate $E_{\mathrm{sbs}}^{\circ }$ value of 3.21%. This range of uncertainties at initial condition generates a range of scale-by-scale errors in the adult ($E_{\mathrm{sbs}}^{f}$) of 11.3% to 20.9%. Hence, the imprecision in color measurement of juveniles is sufficient to explain most, if not all, of the remaining residual unpredictability (15.1%) of the actual adult lizard patterns.

#### Residual unpredictability in other species

**Figure 7:**
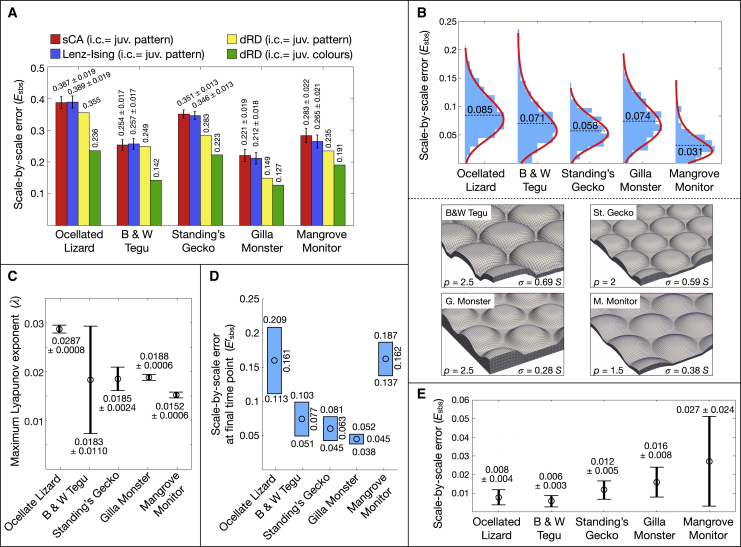
Residual unpredictability and measurement uncertainties (A) Scale-by-scale errors (mean ± SD) of adult patterns simulated with different stochastic and RD models in five lizard species: green columns represent the residual unpredictability of the RD model with juvenile (juv.) colors at initial condition (i.c). (B) Top: distribution and mean (dotted lines) of scale-by-scale error caused by heterogeneity in skin thickness variation (distributions generated as in Figure 5D) in five species of lizards. Lower panels show the reference hexagonal lattices of 3D super-Gaussian bumps with optimized p and σ (p = 1 and σ = 0.28*S* for ocellated lizard). S = mean distance among neighboring scales on a real skin patch of the corresponding species. Heterogeneity in skin thickness variation generates a mean unpredictability of 8.5%, 7.1%, 5.8%, 7.4%, and 3.1% in ocellated lizard, tegu, gecko, Gila monster, and monitor, respectively. (C) Mean and SD of Lyapunov exponent (computed as in Figure S4) for five species of lizards. (D) Range (circles indicate middle of ranges) of scale-by-scale error (at adult time point) obtained with simulations initiated with juvenile colors differing by a small amount $E_{\mathrm{sbs}}^{\circ }$ within the range $\left[\left\|C^{\mathrm{mode}}-C^{\mathrm{ref}}\right\|,\left\|C^{\mathrm{median}}-C^{\mathrm{ref}}\right\|\right]$, where $C^{\mathrm{ref}}$, $C^{\mathrm{mode}}$, and $C^{\mathrm{median}}$ are the mean, mode, and median albedos of juvenile colors. Uncertainties in juvenile color measurements generate a mean unpredictability of 16.1%, 7.7%, 6.3%, 4.5%, and 16.2% in ocellated lizard, tegu, gecko, Gila monster, and monitor, respectively. (E) RD is robust to parameters variation in all five species: scale-by-scale error (mean ± SD) is computed from 5,000 simulations with parameters uniformly sampled in a range covering ±10% of the RD parameter normalization factor estimated during Bayesian optimization.

Below, we investigate whether these results in ocellated lizards can be generalized to the process generating largely different adult patterns in four other species (Figure 1A): the interrupted black banding of the Argentine black and white tegu, the low-contrast highly labyrinthine pattern of the Standing’s day gecko, the large black-and-orange meanders of the Gila monster, and the yellow-on-black speckles of the mangrove monitor. Remarkably, as in the ocellated lizard, RD modeling generates a substantial gain (over stochastic models) in scale-by-scale predictability of observed adult patterns in these four additional species. Indeed, starting from the juvenile thresholded (yellow/orange versus black) pattern, RD systematically generates simulated adult patterns (yellow spots in Figure 3) with a scale-by-scale error statistically smaller than those obtained with the stochastic models (Figure 7A, yellow bar). Similarly again to the ocellated lizard, the gain in predictability by the RD model substantially increases in amplitude and statistical significance when using the juvenile (un-thresholded) real colors as initial condition, leaving a residual scale-by-scale unpredictability (Figure 7A, green bar) of 14.2% for the black and white tegu, 22.3% for the Standing’s gecko, 12.7% for the Gila monster, and 19.1% for the mangrove monitor. Using 3D simulations with heterogeneous skin geometry variation (Figure 7B) and Lyapunov spectrum analyses (Figures 7C and 7D), we show that the residual error in all species is explained by a combination of uncertainty in skin thickness spatial distribution and in color measurement at initial condition: for the ocellated lizard, the mean of the scale-by-scale errors due to uncertainties in skin geometry variation and juvenile color measurement (Figures 7B and 7D) is 8.5% and 16.1%, summing up to 24.6%, i.e., close to the total residual error of 23.6% (Figure 7A). For the tegu, Standing’s gecko, Gila monster, and mangrove monitor, these numbers are 7.1% + 7.7% ≈ 14.2%, 5.8% + 6.3% ≉ 22.3%, 7.4% + 4.5% ≈ 12.7%, and 3.1% + 16.2% ≈ 19.1%, respectively. Only for Standing’s gecko, the means of geometry-induced and of color-induced errors do not approximately sum up to the observed residual error. Although it could be argued that the latter is compatible with the ranges of estimated errors (upper bounds are 14% and 8.1% for geometry- and color-induced errors, respectively), the result above prompted us to further test the validity of the current RD model for describing the Standing’s gecko skin color patterning process. Indeed, performing Gaussian mixture model clustering with 2–6 clusters (using a cluster-validation approach^24^ to evaluate their relative qualities) identified that the optimal number of color clusters is 2 for all individuals of all species, except Standing’s gecko for which it is 3 or 4, depending on the individual. Hence, description of the color change process in Standing’s gecko is likely to require a more complex RD model in which more than two steady-state colors are emerging.

#### Robustness of the RD model

Finally, we conduct in all species a sensitivity analysis in order to estimate how much perturbations of the RD parameters values would affect the scale-by-scale error. To this end, we perform in each species 5,000 “impaired” simulations in which perturbed, rather than optimal, parameter values are used: for each simulation, parameter values are jointly sampled from a uniform distribution covering 20% (i.e., ±10%) of the RD parameters normalization factor (in the covariance function; Equation 32 in STAR Methods) estimated during Bayesian optimization. We then compute, for each perturbed simulation, its scale-by-scale error in comparison with the unperturbed simulation (i.e., with optimal RD parameters) and fit a gamma distribution to the resulting histogram of errors, from which a mean and standard deviation are computed. These analyses indicate that simulations with suboptimal RD parameter values generate smaller mean scale-by-scale errors (0.6% < $E_{\mathrm{sbs}}^{f}$ < 2.8%; Figure 7E) than uncertainties in skin geometry and in juvenile colors (Figures 7B and 7D).

## Discussion

### On evolutionary convergence

Our analyses reveal that two connected spectacular phenotypic characters associated to skin color, scale-by-scale color patterns and cellular automaton dynamics of patterning, have evolved independently in multiple divergent lineages of squamates (Figure 1A). This conclusion has important consequences for our understanding of phenotypic evolution in a developmental context. Indeed, the sequence of events that led to such convergences is, in principle, easy to understand: all that is needed for both phenotypic characters to emerge is that a Turing instability mechanism of skin color patterning occurs in a skin domain with a periodic thickness (here, caused by skin scales). Note that two additional requirements must be met. First, the intrinsic self-organizational Turing length scale of the color pattern should be larger than the length scale of the thickness period. Second, the relative timings of scale development and of skin color patterning are important. In principle, scale-by-scale coloration could occur with both scales developing before or after skin color patterning. Indeed, even if color patterning has reached steady state before the onset of scale development, the pattern should “snap” to the position of scales if the dynamical system of chromatophore interactions remains active. On the other hand, if the color pattern gets fixed (because of the arrest of the underlying microscopic dynamical system) before emergence of scales, then the newborn will not exhibit a scale-by-scale color pattern. Such a situation is observed in ball pythons (*Python regius*). We therefore conjecture that convergences and reversals of these two phenotypic characters, hence, the existence of closely related species differing by the presence/absence of scale-by-scale skin color and/or CA dynamics, can easily occur through heterochrony, i.e., by changes in the relative timings or rates of skin scales development and skin color patterning.

### Prediction of individual scale-by-scale patterns and their neighborhood statistics

Our results show that, in the case of scale-by-scale skin color patterning, simple statistical (such as sCA and Lenz-Ising) models capture well enough the underlying non-linear dynamical system to predict, with high precision, the time evolution of the pattern statistical features. Using the assumption that the functions of skin color patterns are associated to their statistical features,^15^ such as their length scale or their general “look” (e.g., spots versus stripes versus labyrinths), we argue that statistical and RD numerical models can capture most of the functionally relevant behavior of these specific morphogenetic self-organizational systems.

Conversely, we show that (1) the improvement of predictability of the exact scale-by-scale dynamics of individual animals in species exhibiting vastly different color patterns is large for deterministic RD over simpler statistical models and (2) the residual error in exact scale-by-scale color predictability is explained mostly, if not entirely, by uncertainties in skin geometry variation and in color measurements at the initial (juvenile) condition. One could argue that the higher efficiency of RD is due to the incorporation of a large number of parameters (overfitting) and/or to fine-tuning of these parameters. The overfitting argument could hold for the Lenz-Ising but not for the sCA model as the former includes only two parameters (J and B), but the latter incorporates 16 parameters, all of which are optimized in our analyses. Remarkably, parameter fine-tuning can also be ruled out as an explanation for the efficiency of RD. Indeed, the sensitivity analysis reported here (Figure 7E) indicates that the high performance of RD to predict actual scale-by-scale patterns is robust to RD model parameter perturbations, hence, does not require parametrizing the system down to its many nanoscopic/microscopic variables.^18^

In addition, iterative addition of RD model parameters during Bayesian optimization (Table S1) identified the necessity to jointly optimize only 3–5 parameters (Table S2) among a total of 16 (all others are set to the values of Manukyan et al.^12^). This result indicates that the values of the remaining parameters either cannot be optimized beyond those estimated in Manukyan et al.,^12^ or are somewhat unimportant. Examples of the latter are evident in the pairwise parameter gradient plots of Figure S5: e.g., $c_{w}$ and $c_{2}$ affect the pattern much more than $c_{7}$ (cf. 3rd and 6th graphs on the first line of gradient plots) and, similarly, the pattern is very sensitive to $c_{3}$ and relatively insensitive to $c_{u}$ (1st column, 5th row). Note also that obtaining a given low-error pattern depends on the joint optimization of multiple parameters such that single-parameter values within versus among species should not be over-interpreted. For example, despite that optimized values of the growth parameter q (Table S2) differ more between black and white tegus SM1 and SM2 than between SM2 and VI1 (a mangrove monitor lizard), simulating the adult pattern of SM2 using the set of optimized parameter values from SM1 (or vice versa) generates an error ($E_{16D}$ value) smaller than the difference of nearest-neighbor configuration statistics between the two tegu individuals (Table S3). In other words, the joint set of optimized parameters of SM1 or of SM2 generate tegu-specific patterns, whereas the set of parameters of VI1 does not (Table S3). A similar comparison (Table S4) is performed for one ocellated lizard (TL1) with an optimized value of the parameter $D_{u,v}$ (Table S2), closer to that of a tegu (SM1) than to that of another ocellated lizard (TL2). Hence, interpreting RD parameters in isolation is difficult because the RD model is phenomenological rather than built bottom-up from quantified molecular or cellular processes.

In conclusion, the superior efficiency of RD over stochastic models in describing the scale-by-scale skin color patterning process in multiple divergent species of lizards is due to intrinsic properties of RD, including the ability to exploit mesoscopic information such as (1) continuous character states (here, scale colors) and (2) the implicit relation between the pattern length scale and geometrical parameters (here, growth and scale/edge geometries). In other words, spatial discretization of the RD model and/or reduction of its spatial dimensions (from 3D to 2D) do not affect much its efficiency in predicting scale-by-scale patterns, whereas discretization of states (to two colors) in the sCA and Ising models, as well as the inability of these approaches to incorporate diffusion and growth, generate a larger negative impact.

## STAR★Methods

### Key resources table

**Table:** 

REAGENT or RESOURCE	SOURCE	IDENTIFIER
**Biological samples**		

Skin biopsies for HREM	University of Geneva	N/A

**Experimental models: Organisms/strains**		

2 individuals of *T. lepidus*	University of Geneva	LLEP_00038; LLEP_00066
2 individuals of *S. merianae*	University of Geneva	TMER_0001; TMER_0002
4 individuals of *H. suspectum*	University of Geneva	HSUS_001; HSUS_002; HSUS_003; HSUS_004
2 individuals of *V. indicus*	University of Geneva	VIND_0002; VIND_0003
2 individuals of *P. standingi*	University of Geneva	PSTA_00021; PSTA_00022

**Software and algorithms**		

MATLAB R2021a (version 9.10)	https://www.mathworks.com/products/matlab.html	N/A
MATLAB Image Processing Toolbox (version 11.3)	https://www.mathworks.com/products/matlab.html	N/A
MATLAB Statistics and Machine Learning Toolbox (version 12.1)	https://www.mathworks.com/products/statistics.html	N/A
MATLAB Parallel Computing Toolbox (version 7.4)	https://www.mathworks.com/products/parallel-computing.html	N/A

### Resource availability

#### Lead contact

Further information and requests for resources and material should be directed to and will be fulfilled by the lead contact, Michel C. Milinkovitch (Michel.Milinkovitch@unige.ch).

#### Materials availability

This study did not generate new materials.

### Experimental model and subject details

Ocellated lizards, Argentinian tegus, Gila monsters, mangrove monitors and Standing's day geckos are housed in Milinkovitch and Tzika’s laboratory, Department of Genetics and Evolution, University of Geneva, Switzerland. Maintenance of, and experiments on animals were approved by the Geneva Veterinary Cantonal authorities (authorizations GE/82/14, GE/169/17 and GE24/33145) and performed according to the Swiss law. These guidelines meet international standards.

### Method details

#### Animal scanning and identification of the boundaries and colors of skin scales

Animals were scanned with R^2^OBBIE-3D, a robotic system^19^ integrating a six-axis robotic arm, a 36-megapixel digital single-lens reflex color camera (Nikon D810) and an illumination basket of 30 high-intensity light-emitting diodes (LEDs). For each position of the camera, 30 images were taken (each with a different LED, *i.e.*, a different lighting direction) and combined to infer the normal map of the corresponding 3D surface with the photometric stereo (PS) approach.^20^ Depending on the animal size at the time of scanning (they were scanned 14 to 45 times over a period of 2 to 4 years starting from the juvenile stage), 90 to 780 images were taken as the back and flanks were covered by using 3 to 26 different camera positions. The corresponding multiview-PS approach generated 3D models with a resolution of >400 pixels per scale, allowing for the micro-geometry reconstruction of these skin appendages, a pre-requisite for automated identification of skin scales described below. One advantage of the PS approach is that it produces both the normal and the albedo of each pixel. We stored the normal vectors of each PS patch as a 16-bits RGB color image in which each channel contains a component of the normal vector linearly transferred from range (−1,1) to (0,65535).

We then used the normal map to compute the mean curvature of the skin surface, parametrizing the surface $\Phi :R^{2}\to R^{3}$ with the variables u and v:

(Equation 1) $$\Phi \left(u,v\right)=\left(\begin{array}{l} x(u,v)\\ y(u,v)\\ z(u,v) \end{array}\right)$$

where $x=u,\;y=v,\;z=z\left(u,v\right)$. Normals to the surface are then computed as

(Equation 2) $$n=\frac{\Phi_{u}\times \Phi_{v}}{\left\|\Phi_{u}\times \Phi_{v}\right\|}$$

where $\Phi_{u}=\frac{\partial \Phi }{\partial u}$ and $\Phi_{v}=\frac{\partial \Phi }{\partial v}$. Substituting Equation 1 into Equation 2 yields

(Equation 3) $$n=\frac{1}{\sqrt{z_{u}^{2}+z_{v}^{2}+1}}\left(\begin{array}{l} -z_{u}\\ -z_{v}\\ 1 \end{array}\right)$$

where $z_{u}=\frac{\partial z}{\partial u}$ and $z_{v}=\frac{\partial z}{\partial v}$. If the normal vector is $n=\left(\begin{array}{l} n_{1}\\ n_{2}\\ n_{3} \end{array}\right)$, according to Equation 3, we can write $z_{u}=-n_{1}/n_{3},\;z_{v}=-n_{2}/n_{3}$, we can compute $\Phi_{u}$ and $\Phi_{v}$ as

(Equation 4) $$\Phi_{u}=\left(\begin{array}{c} 1\\ 0\\ -\frac{n_{1}}{n_{3}} \end{array}\right),\Phi_{v}=\left(\begin{array}{c} 0\\ 1\\ -\frac{n_{2}}{n_{3}} \end{array}\right)$$

and we can write

(Equation 5) $$\Phi_{uu}=\left(\begin{array}{c} 0\\ 0\\ -\frac{\partial }{\partial u}(\frac{n_{1}}{n_{3}}) \end{array}\right),\Phi_{vv}=\left(\begin{array}{c} 0\\ 0\\ -\frac{\partial }{\partial u}(\frac{n_{2}}{n_{3}}) \end{array}\right),\Phi_{uv}=\left(\begin{array}{c} 0\\ 0\\ -\frac{\partial }{\partial u}(\frac{n_{2}}{n_{3}}) \end{array}\right)$$

where $\Phi_{uu}=\frac{\partial^{2}\Phi }{\partial u^{2}}$, $\Phi_{vv}=\frac{\partial^{2}\Phi }{\partial v^{2}}$, and $\Phi_{uv}=\frac{\partial^{2}\Phi }{\partial u\partial v}$.

We then compute the mean curvature by using the first and second fundamental form, $F_{1}$ and $F_{2}$.

$$F_{1}=\left[\begin{array}{cc} E & F\\ F & G \end{array}\right],F_{2}=\left[\begin{array}{cc} L & M\\ M & N \end{array}\right]$$

in which

(Equation 6) $$\begin{array}{l} E=\Phi_{u}\cdot \Phi_{u},\;F=\Phi_{u}\cdot \Phi_{v},\;G=\Phi_{v}\cdot \Phi_{v}\\ L=\Phi_{uu}\cdot n,\;\;M=\Phi_{uv}\cdot n,\;N=\Phi_{vv}\cdot n \end{array}$$

After substituting the derivatives of Φ from Equations 4 and 5 into Equation 6, we have

(Equation 7) $$\begin{array}{l} E=1+{\left(\frac{n_{1}}{n_{3}}\right)}^{2},\;F=\frac{n_{2}n_{1}}{n_{3}^{2}},\;G=1+{\left(\frac{n_{2}}{n_{3}}\right)}^{2}\\ L=-\frac{\partial }{\partial u}\left(\frac{n_{1}}{n_{3}}\right)n_{3},\;M=-\frac{\partial }{\partial v}\left(\frac{n_{1}}{n_{3}}\right)n_{3},\;N=-\frac{\partial }{\partial v}\left(\frac{n_{2}}{n_{3}}\right)n_{3} \end{array}$$

The mean curvature H is then computed as

(Equation 8) $$H=\frac{EN-2FM+GL}{2\left(EG-F^{2}\right)}$$

By substituting Equation 7 into Equation 8, we compute the mean curvature (from the normal map) as

$$H=-\frac{n_{3}}{2}\left(n_{3}^{2}+n_{1}^{2}\right)\frac{\partial }{\partial v}\left(\frac{n_{2}}{n_{3}}\right)+n_{3}n_{2}n_{1}\frac{\partial }{\partial v}\left(\frac{n_{1}}{n_{3}}\right)-\frac{n_{3}}{2}\left(n_{3}^{2}+n_{2}^{2}\right)\frac{\partial }{\partial u}\left(\frac{n_{1}}{n_{3}}\right)$$

Figures 1B and 1C show the normal map and the mean curvature for a rectangular patch of skin of an ocellated lizard (TL1) at different post-hatching developmental stages. We then apply both a Gaussian and a Median filter on the mean curvature field to remove very short wavelength noises while preserving the sharpness of scales boundaries. Note that, as the average scale size in pixels is about s, we find that smoothing is efficiently applied by the Gaussian and Median filters with a variance σ=s/10 and window size $w={\left(s,s\right)}^{T}$, respectively. Figure 1C shows the mean curvature after applying the two filters. Each local minimum in the filtered curvature field indicates an individual scale. However, the actual centroid of the corresponding scale is not necessarily located at this minimum because scales in all the species investigated (except Standing’s gecko) are slightly asymmetrical and tend to have their lowest curvature slightly displaced distally (*i.e.*, caudally). Hence, we use the watershed algorithm to segment the filtered mean curvature grayscale images with the darkest regions representing the local minima. Although the resulting segmentation of scales is approximate, the centroids of the watershed segments better correspond to the actual center of scales. Proper scale boundaries (red lines in Figure 1C) are then identified by applying Voronoi tessellation on the centroids of the watershed segments.

After identifying scale borders as described above, scale colors are determined by averaging color across pixels within each scale. Note that pixels too close to scales boundaries (distance < s/10 ) are excluded to avoid artifactual shading due to the high curvature of scale borders.

#### Scale matching

In order to trace, across time points, all scales in the Voronoi meshes generated for each time-point, we first attempt to match all possible pairs of Voronoi meshes. This is achieved by finding for each scale an affine transformation (allowing for translations, rotations, scaling and shear) which transforms the scale centers between two Voronoi meshes at times *r* and *t*. The affine matrix *M* in 2D transforms the target coordinates $\left(\begin{array}{c} x_{t}\\ y_{t} \end{array}\right)$ into the reference coordinate $\left(\begin{array}{c} x_{r}\\ y_{r} \end{array}\right)$ according to

$$\left(\begin{array}{c} x_{r}\\ y_{r}\\ 1 \end{array}\right)=M\left(\begin{array}{c} x_{t}\\ y_{t}\\ 1 \end{array}\right)$$

where M is a 3 × 3 matrix which contains 6 variable coefficients as

$$M=\left(\begin{array}{ccc} m_{11} & m_{12} & m_{13}\\ m_{21} & m_{22} & m_{23}\\ 0 & 0 & 1 \end{array}\right)$$

If we know at least three matching points between the reference and the target spaces, we can find M as

$$M=\left(\begin{array}{ccc} x_{r1} & x_{r2} & x_{r3}\\ y_{r1} & y_{r2} & y_{r3}\\ 1 & 1 & 1 \end{array}\right){\left(\begin{array}{ccc} x_{t1} & x_{t2} & x_{t3}\\ y_{t1} & y_{t2} & y_{t3}\\ 1 & 1 & 1 \end{array}\right)}^{-1}$$

If we use >3 points to compute M, we obtain an over-determined system yielding a least-square approximation of the affine transformation. After initiating the matching algorithm by manually selecting the centers of at least 3 scales in all time points, the first iteration consists into computing an affine transformation for each scale (independently for each pair of time points) that is a first neighbor of any of the manually-selected scales, *i.e.*, affine matrices are computed independently for each neighbor scale. The center of each of these newly-considered scales is then transferred from the source to the target space by using the corresponding affine matrix. If the distance between a transferred point and a scale center in the target lattice is smaller than ε pixels (*i.e.*, 0.05 times the average distance between neighboring scales), the two centers are registered as a match between the source and target lattices. Once all transferred points have been tested, the patch in the source lattice is updated by adding the new matches. The process is then iterated by finding all first neighbors of scales in the updated patch and computing the corresponding affine transformations. The affine transformation for each new scale considers at least 3 and at most 10 closest neighbors already matched. Note that points that have been transferred but not matched at iteration i will necessarily be first-neighbors of the updated patch and will therefore be transferred again at iteration *i*+1. These points will be more likely to find a match in the target lattice because the corresponding affine matrix will involve more neighbors already matched. The matching algorithm stops when no new match is found.

After matching all pairs of time points, we combine all pairwise matches into a global graph that we term a ‘space-time Voronoi network’, in which nodes are scales (each scale receives a unique label at each time-point) and edges are matches. Each scale for which the number of nodes in the global graph equals the number of time-points is selected for the next step. In other words, the selected connected components should represent the scales for which at least one match exists in each time-point. The final patch of matched scales generated by the algorithm is a lattice with a jagged boundary and possibly some missing elements inside. More specifically, remaining unmatched points correspond to real scales that cannot be matched with the use of an affine transformation. At this stage, we find a very small proportion of these errors (average = 5%) which are then easily corrected individually by manually identifying the corresponding scale in one time-point such that the matches are automatically generated at all other time-points using local affine transformations. Next, we correct for errors in connectivity. According to our observations, the number of scales as well as their connectivity are invariant in time. Hence, the approximately 10% mismatch in connectivity that we observe between pairs of time-points are errors that should be corrected. For that purpose, we generate a unified space-time Voronoi network by averaging (after correcting for scaling, translation and rotation among time-points) the centers of each scale across time points. We exclude shear and anisotropic scaling because they would transform the Voronoi network topology in favor of the reference time-point. Given these constraints, we can write the transformation as

(Equation 9) $$\left(\begin{array}{c} x_{p}\\ y_{p} \end{array}\right)=\left(\begin{array}{cc} s\;\cos\left(\theta \right) & -s\;\sin\left(\theta \right)\\ s\;\sin\left(\theta \right) & s\;\cos\left(\theta \right) \end{array}\right)\left(\begin{array}{c} x_{t}^{'}\\ y_{t}^{'} \end{array}\right),\left(\begin{array}{c} x_{t}^{'}\\ y_{t}^{'} \end{array}\right)=\left(\begin{array}{c} x_{t}\\ y_{t} \end{array}\right)+t$$

in which s is the isotropic scaling factor, θ is the rotation angle, $t=\left(\begin{array}{c} t_{x}\\ t_{y} \end{array}\right)$ is the translation vector and subscript _*t*_ and _*p*_ denote the coordinates of target scales before and after transformation, respectively. The translation vector t can be easily found from the centroids of the scales as

$t=\left(\begin{array}{c} \bar{x_{r}}-\bar{x_{t}}\\ \bar{y_{r}}-\bar{y_{t}} \end{array}\right)$, where subscripts _*r*_ denotes the coordinates of the reference scales and $\bar{x}=\frac{1}{N}\sum_{i=1}^{N}x_{i}$ denotes to the average in which N is the number of scales. In order to find θ and *s*, we use the least-squares approach to minimize the error function defined as

(Equation 10) $$E=\sum_{i=1}^{N}\left({\left(x_{r,i}-x_{p,i}\right)}^{2}+{\left(y_{r,i}-y_{p,i}\right)}^{2}\right)$$

Substituting Equation 9 into Equation 10 and solving for ∂E/∂θ=0 and ∂E/∂s=0, we get

$$\theta =\arctan\left(\frac{\bar{y_{r}x_{t}^{'}}-\bar{x_{r}y_{t}^{'}}}{\bar{x_{r}x_{t}^{'}}+\bar{y_{r}y_{t}^{'}}}\right),\;s=\frac{\cos\left(\theta \right)\left(\bar{x_{r}x_{t}^{'}}+\bar{y_{r}y_{t}^{'}}\right)+\sin\left(\theta \right)\left(\bar{y_{r}x_{t}^{'}}-\bar{x_{r}y_{t}^{'}}\right)}{\bar{{x_{t}^{'}}^{2}}+\bar{{y_{t}^{'}}^{2}}}$$

By knowing *θ*, *s* and ***t***, we can use Equation 9 to transfer all time-points to the reference space and then take, for each scale, its across-time-point average position. The averaged scale centers are then used to generate the final space-time Voronoi network (including color of scales) for the corresponding animal. Figure S6 shows the resulting matched scales for 25 time-points of the ocellated lizard TL1.

#### Stochastic Cellular Automaton

First, we label all the scales in the Voronoi lattice by one of the nearest-neighbor configurations in the set

$$C=\left\{\left(S,i\right)|S\in \left\{\mathrm{Green},\;\mathrm{Black}\right\},i\in \left\{0,1,2,3,4,5,6,7\right\}\right\}$$

where S indicates the state (green or black) of the scale and i denotes the number of isochromatic neighbors. We assume that the probability p of a scale changing color in the space-time network (Figure 1F) only depends on its nearest-neighbor configuration, *i.e.*, p=p(S,i), such that the sCA model can be defined with 2×8=16 parameters. As frequencies of rare color changes, and/or of changes that involve rare nearest-neighbor configurations, are poor estimates of the corresponding color-change probabilities p, we use Bayesian inference to compute the expectation E[p] of these probabilities. Assuming that color change follows a Binomial distribution with probability of k successes from n trials, the posterior probability of p (assuming flat priors) is

$$f_{p|k,n}=\left(n+1\right)\left(\begin{array}{c} n\\ k \end{array}\right)p^{k}{\left(1-p\right)}^{n-k}$$

with the maximum of that posterior distribution function positioned at

$$p_{MAP}=\arg\max_{p}\;f_{p|k,n}=\frac{k}{n}$$

*i.e.*, at the corresponding observed color-change frequency. On the other hand, the expectation of p is:

$$E\left[p\right]=\frac{k+1}{n+2}$$

The sCA rules are obtained simply by summing up trials and successes across time-points and deriving probabilities for each of the 16 possible configurations. Note that, during numerical simulations, we adapt the number of sCA iterations such that the cumulative sum of color flips between initial and final time-points is approximately equal to the number of flips observed in the corresponding real animal. As an example, the time-evolution of neighborhood statistics in two ocellated lizard individuals (TL1 and TL2) are shown in Figures S1A and S2A.

#### Reaction-diffusion

Following the notation of Manukyan et al.^12^ and Fofonjka and Milinkovitch,^14^ we use a RD model with three components u,v,w such that the three coupled partial differential equations (PDEs) can be written in matrix form as

(Equation 11) $$\partial_{t}u=F\left(u\right)-\mathrm{cu}+D\nabla^{2}u$$

in which

$$u=\left(\begin{array}{c} u\\ v\\ w \end{array}\right),\;F\left(u\right)=\left(\begin{array}{c} F\left(u,v,w\right)\\ G\left(u,v,w\right)\\ H\left(u,v,w\right) \end{array}\right),\;c=\left(\begin{array}{ccc} c_{u} & 0 & 0\\ 0 & c_{v} & 0\\ 0 & 0 & c_{w} \end{array}\right),\;D=\left(\begin{array}{ccc} D_{u} & 0 & 0\\ 0 & D_{v} & 0\\ 0 & 0 & D_{w} \end{array}\right)$$

and the Laplacian operator can be written in the compact form

(Equation 12) $$\nabla^{2}u=\nabla \cdot \left(\nabla u\right)=\left(\begin{array}{c} \partial_{xx}^{2}u+\partial_{yy}^{2}u+\partial_{zz}^{2}u\\ \partial_{xx}^{2}v+\partial_{yy}^{2}v+\partial_{zz}^{2}v\\ \partial_{xx}^{2}w+\partial_{yy}^{2}w+\partial_{zz}^{2}w \end{array}\right)$$

Note that the non-linearity of the reaction terms is due to their step-wise function form:

$$F\left(u,v,w\right)=\{\begin{array}{ccc} 0 & : & c_{1}v+c_{2}w+c_{3}<0\\ c_{1}v+c_{2}w+c_{3} & : & 0\leq c_{1}v+c_{2}w+c_{3}\leq F_{\max}\\ F_{\max} & : & F_{\max}<c_{1}v+c_{2}w+c_{3} \end{array}$$

(Equation 13) $$G\left(u,v,w\right)=\{\begin{array}{ccc} 0 & : & c_{4}u+c_{5}w+c_{6}<0\\ c_{4}u+c_{5}w+c_{6} & : & 0\leq c_{4}u+c_{5}w+c_{6}\leq G_{\max}\\ G_{\max} & : & G_{\max}<c_{4}u+c_{5}w+c_{6} \end{array}$$

$$H\left(u,v,w\right)=\{\begin{array}{ccc} 0 & : & c_{7}u+c_{8}v+c_{9}<0\\ c_{7}u+c_{8}v+c_{9} & : & 0\leq c_{7}u+c_{8}v+c_{9}\leq H_{\max}\\ H_{\max} & : & H_{\max}<c_{7}u+c_{8}v+c_{9} \end{array}$$

##### 3D continuous RD (3D-cRD)

The Laplacian operator in Equation 12 is defined in the Cartesian space. We then rewrite the Laplacian operator in curvilinear 3D space (accurately representing the skin geometry) as follows. We define curvilinear space coordinates $y_{i}$, parametrized by Cartesian space coordinates, $x_{i}$, as

$$y_{i}=y_{i}\left(x_{1},x_{2},x_{3}\right)$$

in which i=1,2,3 is the dimension index. In order to find the equivalent operator in the curvilinear space, we first rewrite the gradient operator as

$$\nabla u=\frac{\partial u}{\partial x_{i}}e_{i}=\frac{\partial u}{\partial y_{i}}b^{i}$$

in which $e_{i}$ are the basis vectors in the Cartesian system and

$$b^{i}=\nabla y_{i}=\frac{\partial y_{i}}{\partial x_{j}}e_{j}=b_{j}^{i}e_{j}$$

is the covariant basis in the curvilinear system.

Then, the Laplacian of u reads

(Equation 14) $$\begin{array}{ccc} \nabla^{2}u & = & \nabla \cdot \left(\nabla u\right)\;=\;\nabla \cdot \left(\frac{\partial u}{\partial y_{i}}b^{i}\right)\\ & = & \nabla \left(\frac{\partial u}{\partial y_{i}}\right)\cdot b^{i}+\frac{\partial u}{\partial y_{i}}\nabla \cdot b^{i}\\ & = & \frac{\partial^{2}u}{\partial y_{j}\partial y_{i}}b^{j}\cdot b^{i}+\frac{\partial u}{\partial y_{i}}\frac{\partial b_{j}^{i}}{\partial y_{k}}\frac{\partial y_{k}}{\partial x_{j}}\\ & = & \frac{\partial^{2}u}{\partial y_{j}\partial y_{i}}b^{j}\cdot b^{i}+\frac{\partial u}{\partial y_{i}}\frac{\partial b_{j}^{i}}{\partial y_{k}}b_{j}^{k} \end{array}$$

The covariant basis components, $b_{i}^{j}$, appearing in Equation 14 are computed for all nodes in the structured grid. The nodes located at the grid borders are named ‘boundary nodes’ whereas all others are ‘interior nodes’. The latter are assumed to be at the center of a 3×3×3 stencil for which the finite-difference approximation is computed and the discrete Laplacian operator is written in a matrix form using the local indices of the nodes in the stencil. In order to approximate the covariant basis components of a node, we take into account the $x_{i}$ and $y_{i}$ variations of all 27 neighbor nodes in the corresponding stencil. More precisely, the metric tensor components are the slope of the lines calculated using the least-square linear regression on the 27 data points. For boundary nodes, there are 22 possible different stencils (all with <27 nodes), but the least-square linear regression remains valid for these boundary nodes. Then, we use finite-difference formulas to approximate the first, second and cross derivatives appearing in Equation 14. Finally, Equation 11 is time integrated using the Euler backward scheme. Then, the resulting linear system is solved with a stabilized variant of the Bi-conjugate gradient method (BiCGSTAB^25^).

##### 2D continuous RD (2D-cRD)

As the skin domain thickness (h in the Z dimension) is much smaller than its size in the two other spatial dimensions, it is reasonable to neglect variations of RD variables across Z. This assumption enables reducing the model from 3D to 2D by integrating in the Z direction^14^ the RD equations (Equation 11) to give

$$\partial_{t}\bar{u}=F\left(\bar{u}\right)-c\bar{u}+\frac{1}{\bar{h}}\nabla \cdot \left(h\left(x\right)D\nabla \bar{u}\right)$$

in which $\bar{u}=\frac{1}{h}\int_{z_{\circ }}^{z_{\circ }+h}udz$ and $\bar{h}=\frac{1}{A_{\mathrm{skin}}}\int_{A_{\mathrm{skin}}}h\left(x\right)dA$.

Using a position-dependent diffusion coefficient $D\left(x\right)=h\left(x\right)/\bar{h}D$, we can rewrite the reduced-order RD equations as

$$\partial_{t}\bar{u}=F\left(\bar{u}\right)-c\bar{u}+\nabla \cdot \left(D\left(x\right)\nabla \bar{u}\right)$$

As we are building a 2D model, we replace hereafter $\bar{u}$ by u. We can further simplify the model by assuming that scales are prismatic (*i.e.*, thickness is reduced only at discrete scale borders). In 2D, this is translated into taking into account the reduction of skin thickness through scaling of the continuous RD diffusion coefficients (by a factor *P*) at the one-dimensional edges of the 2D scales,^12^ *i.e.*, we introduce a position-dependent diffusion coefficient in the 2D model, with the diffusion matrix reading

$$D\left(x\right)=\{\begin{array}{cc} PD & x\in \text{scale boundary}\\ D & \text{else} \end{array}$$

Then, similar to the 3D-cRD case above, we use the finite-difference method to discretize the diffusion term. For that purpose, we consider that the physical domain is discretized into a square grid with spacing *ε*. As both the physical and computational domains are equivalent square grids, the metric tensor simplifies into a unit tensor. Similarly to Manukyan et al.,^12^ the 2D-cRD equations become

(Equation 15) $$\frac{du_{i}}{dt}=F\left(u_{i}\right)-cu_{i}+\frac{1}{\varepsilon^{2}}\sum_{j}D\left({\bar{x}}_{ij}\right)\left(u_{j}-u_{i}\right)$$

where ${\bar{x}}_{ij}=\left(x_{i}+x_{j}\right)/2$ and j represents the neighbors of the i ^th^ node.

##### 2D Discrete RD (2D-dRD)

We derive here the discrete-RD equations by using the finite-volume method for arbitrary polygons. The governing equations (Equation 1) are integrated over the skin domain Ω.

$$\int_{\Omega }\partial_{t}udA=\int_{\Omega }\left[F\left(u\right)-\mathrm{cu}+\nabla \cdot \left(D\left(x\right)\nabla u\right)\right]dA$$

Using the Divergence theorem, we obtain

$$\frac{d}{dt}\int_{\Omega }udA=\int_{\Omega }\left(F\left(u\right)-\mathrm{cu}\right)dA+\int_{\partial \Omega }D\left(x\right)\frac{\partial u}{\partial n}dL$$

Then we write this integral for a single scale labeled by index *i*:

(Equation 16) $$A_{i}\frac{du_{i}}{dt}=A_{i}\left[F\left(u_{i}\right)-cu_{i}\right]+PD\sum_{j}{\left(\frac{\partial u}{\partial n}\right)}_{ij}L_{ij}$$

where $A_{i}=\int_{\Omega_{i}}dA$, $L_{ij}=\int_{\partial \Omega_{i}\cap \partial \Omega_{j}}dL$, and $u_{i}=\frac{1}{A_{i}}\int_{\Omega_{i}}udA$

are the scale area, the length of the edge between scales i and j, and the area-averaged values of component u, respectively. To solve Equation 16, we need to compute ${\left(\partial u/\partial n\right)}_{ij}$, *i.e.*, the diffusion flux exchanged between neighboring scales. Recalling that the scales boundary thickness is *ε*, we can approximate the normal-oriented flux as

$${\left(\frac{\partial u}{\partial n}\right)}_{ij}\approx \frac{u_{j}-u_{i}}{\varepsilon }$$

This approximation implies that the RD component concentrations are changing linearly across interscale edges. Finally, substituting the normal flux into Equation 16, we obtain the discrete RD equations for arbitrary polygonal scales:

(Equation 17) $$\frac{du_{i}}{dt}=F\left(u_{i}\right)-cu_{i}+\frac{PD}{A_{i}\varepsilon }\sum_{j}\left(u_{j}-u_{i}\right)L_{ij}$$

Note that, if we assume a regular hexagonal lattice, with edge length S, we have

$$A_{i}=\frac{3\sqrt{3}}{2}S^{2},\;L_{ij}=S$$

such that Equation 17 simplifies to the discrete equation derived in Manukyan et al.^12^:

(Equation 18) $$\frac{du_{i}}{dt}=F\left(u_{i}\right)-cu_{i}+\frac{2PD}{3\sqrt{3}S\varepsilon }\sum_{j}\left(u_{j}-u_{i}\right)$$

#### Enhanced RD

Here we integrate growth of the animal as well as variation of color state at initial condition.

##### Growth model

We assume growth to change as a logistic function

(Equation 19) $$S\left(\tau \right)=\frac{k_{1}}{exp\left(-k_{2}\tau +k_{3}\right)+1}$$

in which S is the average (across all scales) of edges’ lengths, τ is the growth time variable and $k_{i=1,2,3}$ are the constants determined by fitting the logistic curve to the measurements made on the corresponding real animals. Figure S7A shows the measured scales size as well as the fitted growth functions for the two ocellated lizards (TL1 and TL2). To integrate isotropic growth in the RD equations, we must take into account the different time-scales of the growth *versus* RD processes. Here, we consider that they are related by a rate factor q as

$$q=\frac{dt}{d\tau }$$

in which τ and t denote the time variables of growth and RD processes, respectively. Then, we can rewrite the discrete RD Equation 17 as a function of τ.

(Equation 20) $$\frac{1}{q}\frac{du_{i}}{d\tau }=F\left(u_{i}\right)-cu_{i}+\frac{PD}{A_{i}\left(\tau \right)\varepsilon \left(\tau \right)}\sum_{j}\left(u_{j}-u_{i}\right)L_{ij}\left(\tau \right)$$

in which *ε*, L and A are changing as a functions of τ. Knowing the scales growth function S(τ) from Equation 19, we determine the growth of all lengths and areas in proportion to the reference time-point geometry such that

(Equation 21) $$\varepsilon \left(\tau \right)=\frac{S\left(\tau \right)}{S_{\mathrm{ref}}}\varepsilon_{\mathrm{ref}},\;L_{ij}\left(\tau \right)=\frac{S\left(\tau \right)}{S_{\mathrm{ref}}}L_{ij,\mathrm{ref}},\;A_{i}\left(\tau \right)={\left(\frac{S\left(\tau \right)}{S_{\mathrm{ref}}}\right)}^{2}A_{i,\mathrm{ref}}$$

Here, we assume $\varepsilon_{\mathrm{ref}}=1$ and select the last measured time-point as the reference geometry. Substituting Equation 21 into Equation 20 results in a discrete RD (2D-dRD) model integrating growth:

$$\frac{1}{q}\frac{du_{i}}{d\tau }=F\left(u_{i}\right)-cu_{i}+{\left(\frac{S_{\mathrm{ref}}}{S\left(\tau \right)}\right)}^{2}\frac{PD}{A_{i,\mathrm{ref}}\varepsilon_{\mathrm{ref}}}\sum_{j}\left(u_{j}-u_{i}\right)L_{ij,\mathrm{ref}}$$

Similarly, we can integrate growth into the 2D continuous RD (2D-cRD) model by using dt=qdτ and substituting $\varepsilon \left(\tau \right)=\frac{S\left(\tau \right)}{S_{\mathrm{ref}}}\varepsilon_{\mathrm{ref}}$ into Equation 15 to obtain

$$\frac{1}{q}\frac{du_{i}}{d\tau }=F\left(u_{i}\right)-cu_{i}+{\left(\frac{S_{\mathrm{ref}}}{S\left(\tau \right)}\right)}^{2}\frac{1}{\varepsilon_{\mathrm{ref}}^{2}}\sum_{j}D\left({\bar{x}}_{ij,\mathrm{ref}}\right)\left(u_{j}-u_{i}\right)$$

##### Juvenile initial condition (IC) and color transformation matrix

Determining the initial condition (corresponding to the juvenile pattern) requires transferring color information to the space of RD variables. Here, we detail how to obtain the transformation function. First, we linearize the RD equations close to the homogeneous steady state (HSS). By definition, at HSS the temporal and spatial derivatives in Equation 11 vanish, such that

(Equation 22) $$F\left(u^{*}\right)=cu^{*}$$

where the vector $u^{*}$ corresponds to the RD component concentrations $\left(u^{*},v^{*},w^{*}\right)$ at the HSS. The reaction terms defined in Equation 13 partition the u,v,w space into 9 linear regions. As we assume that our RD system is initiated by values all located in the central region, the reaction terms F, G and H read

(Equation 23) $$\begin{array}{cc} & F\left(u,v,w\right)=c_{1}v+c_{2}w+c_{3}\\ & G\left(u,v,w\right)=c_{4}u+c_{5}w+c_{6}\\ & H\left(u,v,w\right)=c_{7}u+c_{8}v+c_{9} \end{array}$$

Substituting Equation 23 into Equation 22, we have

$$\left(\begin{array}{c} u^{*}\\ v^{*}\\ w^{*} \end{array}\right)={\left(\begin{array}{ccc} -c_{u} & c_{1} & c_{2}\\ c_{4} & -c_{v} & c_{5}\\ c_{7} & c_{8} & -c_{w} \end{array}\right)}^{-1}\left(\begin{array}{c} c_{3}\\ c_{6}\\ c_{9} \end{array}\right)$$

In addition, as we assume that the HSS belongs to the central region, valid values of the parameters $\left(c_{1},\ldots ,c_{9},c_{u},c_{v},c_{w}\right)$ must respect

(Equation 24) $$\begin{array}{cc} & 0<c_{1}v^{*}+c_{2}w^{*}+c_{3}\leq F_{\max}\\ & 0<c_{4}u^{*}+c_{5}w^{*}+c_{6}\leq G_{\max}\\ & 0<c_{7}u^{*}+c_{8}v^{*}+c_{9}\leq H_{\max} \end{array}$$

such that $u^{*}$ is a point in 3D u,v,w space inside the region delimited by the six planes defined by Equation 24. We then linearize the governing 2D-dRD equations on the hexagonal lattice: substituting Equation 23 into Equation 18 yields

(Equation 25) $$\frac{du_{i}}{dt}=\left(\begin{array}{ccc} -c_{u} & c_{1} & c_{2}\\ c_{4} & -c_{v} & c_{5}\\ c_{7} & c_{8} & -c_{w} \end{array}\right)u_{i}+\left(\begin{array}{c} c_{3}\\ c_{6}\\ c_{9} \end{array}\right)+\frac{2P}{3\sqrt{3}S\varepsilon }D\left(\sum_{j}u_{j}-6u_{i}\right)$$

We can then approximate the summation terms in Equation 25 as $\sum_{j}u_{j}=6u^{*}$ and define

$$b=\left(\begin{array}{c} c_{3}\\ c_{6}\\ c_{9} \end{array}\right)+\frac{4PD}{\sqrt{3}S\varepsilon }u^{*},M=\left(\begin{array}{ccc} -c_{u} & c_{1} & c_{2}\\ c_{4} & -c_{v} & c_{5}\\ c_{7} & c_{8} & -c_{w} \end{array}\right)-\frac{4PD}{\sqrt{3}S\varepsilon }$$

to write Equation 25 in a compact form representing a system of linear ordinary differential equations

(Equation 26) $$\partial_{t}u=\mathrm{Mu}+b$$

whose solution describing the time evolution of u is

(Equation 27) $$u\left(t\right)=e^{Mt}\left(u_{\circ }+M^{-1}b\right)-M^{-1}b$$

in which $u_{\circ }$ is the initial condition. We can then use spectral decomposition to rewrite Equation 27 as $M=V\Lambda V^{-1}$, where Λ is the diagonal matrix which contains eigenvalues of M.

$$\Lambda =\left(\begin{array}{ccc} \lambda_{1} & 0 & 0\\ 0 & \lambda_{2} & 0\\ 0 & 0 & \lambda_{3} \end{array}\right)$$

and V is a matrix in which columns are $V=\left(\begin{array}{ccc} v_{1} & v_{2} & v_{3} \end{array}\right)$, *i.e.*, the eigenvectors of M. Then, the exponential of the matrix M can be written as $e^{M}=Ve^{\Lambda }V^{-1}$ which enables us to rewrite Equation 27 as

(Equation 28) $$\begin{array}{ccc} u\left(t\right) & = & Ve^{\Lambda t}V^{-1}\left(u_{\circ }+M^{-1}b\right)-M^{-1}b\\ & = & Ve^{\Lambda t}\left(\begin{array}{c} a_{1}\\ a_{2}\\ a_{3} \end{array}\right)-M^{-1}b\\ & = & e^{\lambda_{1}t}a_{1}v_{1}+e^{\lambda_{2}t}a_{2}v_{2}+e^{\lambda_{3}t}a_{3}v_{3}-M^{-1}b \end{array}$$

whereas $a_{1,2,3}$ are constant coefficients computed as

$$\left(\begin{array}{c} a_{1}\\ a_{2}\\ a_{3} \end{array}\right)=V^{-1}\left(u_{\circ }+M^{-1}b\right)$$

Equation 28 is an approximation of a scale color trajectory in u,v,w space. When t is large enough, the maximum eigenvalue term ($\lambda_{1}$) dominates and the trajectory simplifies to the dominating eigenvector line

(Equation 29) $$u\left(t^{\prime }\right)=v_{1}t^{\prime }-M^{-1}b$$

where $t^{\prime }=e^{\lambda_{1}t}a_{1}$ can take negative or positive values depending on the sign of $a_{1}$ which itself depends on the initial value $u_{\circ }$. If $\lambda_{1}<0$, the system is stable and the trajectory converges to the steady state $u^{*}$, whereas, if $\lambda_{1}>0$, the scale color trajectory is directed towards one of two ‘extremities’ of the dominating eigenvector line, *i.e.*, the 3D points where the line defined by Equation 29 intersects the planes described by Equation 24. Figure S7B shows the color trajectories in u,v,w space starting from different initial conditions. Note that performing this analysis on the RD model integrating growth (see previous section) produces the same dominating eigenvector line.

After performing the linear stability analysis above, we use the dominating eigenvector line Equation 29 to convert the color data of all juvenile scales to u,v,w space (Figure S7C). Indeed, assuming that juvenile scale colors correspond to color variables close to the HSS and vary along the dominating eigenvector line, we construct a transformation matrix that converts color data to u,v,w (and *vice versa*) as follows. First, we convert both juvenile and adult scale colors from RGB to CIELAB color space which expresses color along three axes: perceptual lightness (*L*^∗^; with black at *L*^∗^=0 and white at *L*^∗^=100), unbound green–red opponents (*a*^∗^; with *a*^∗^<0 towards green and *a*^∗^>0 towards red) and unbound blue–yellow opponents (*b*^∗^; with blue towards *b*^∗^<0 and yellow towards *b*^∗^>0). We use the CIELAB color space because it was designed to approximate the nonlinear response of the human eye. We then use the K-mean algorithm to divide color data into two clusters. The cluster centers are denoted by

$$C_{g}={\left(\begin{array}{c} L\\ a\\ b \end{array}\right)}_{g}\mathrm{and}\;C_{b}={\left(\begin{array}{c} L\\ a\\ b \end{array}\right)}_{b}$$

where $L_{g}>L_{b}$.

Using Equations 18 and 24, we compute the two extremities of the distribution line as

$$u_{g}={\left(\begin{array}{c} u\\ v\\ w \end{array}\right)}_{g}\mathrm{and}\;u_{b}={\left(\begin{array}{c} u\\ v\\ w \end{array}\right)}_{b}$$

where $v_{g}>v_{b}$.

We then construct two affine transformation matrices: the first projects every point in color space on the line passing through $C_{g}$ and $C_{b}$ whereas the second transfers the projected points to the line passing through $u_{g}$ and $u_{b}$ in u,v,w space. The two corresponding matrices are denoted by $T_{1}\left(C_{g},C_{b}\right)$ and $T_{2}\left(C_{g},C_{b},u_{g},u_{b}\right)$, respectively. For arbitrary vectors $x,x^{\prime },y$, and $y^{\prime }$, the matrices $T_{1}$ and $T_{2}$ read

$$T_{1}\left(x,x^{\prime }\right)=\left(\begin{array}{cc} e\left(x,x^{\prime }\right)e{\left(x,x^{\prime }\right)}^{T} & \left(I-e\left(x,x^{\prime }\right)e{\left(x,x^{\prime }\right)}^{T}\right)x\\ 0 & 1 \end{array}\right)$$

$$T_{2}\left(x,x^{\prime },y,y^{\prime }\right)=\left(\begin{array}{cc} e\left(y,y^{\prime }\right)e{\left(x,x^{\prime }\right)}^{T}\frac{\left\|y-y^{\prime }\right\|}{\left\|x-x^{\prime }\right\|} & y-\left(e\left(y,y^{\prime }\right)e{\left(x,x^{\prime }\right)}^{T}\frac{\left\|y-y^{\prime }\right\|}{\left\|x-x^{\prime }\right\|}\right)x\\ 0 & 1 \end{array}\right)$$

where $e\left(x,x^{\prime }\right)=\frac{x-x^{\prime }}{\left\|x-x^{\prime }\right\|}$.

In order to allow optimizing the initial condition, all the transferred points are moved closer to the HSS by applying $T_{3}$ which reads

$$T_{3}\left(r,u^{*}\right)=\left(\begin{array}{cc} rI & \left(1-r\right)u^{*}\\ 0 & 1 \end{array}\right)$$

where r is an adjustable parameter ranging between 0 (the point is moved to the HSS) and 1 (the point is not moved). Finally, the color of each juvenile scale is converted to the u,v,w space using the combination of the $T_{1}$, $T_{2}$, and $T_{3}$ matrices (see Figure S7C):

(Equation 30) $$\left(\begin{array}{c} u\\ 1 \end{array}\right)=T_{3}\left(r,u^{*}\right)T_{2}\left(C_{g},C_{b},u_{g},u_{b}\right)T_{1}\left(C_{g},C_{b}\right)\left(\begin{array}{c} C\\ 1 \end{array}\right)$$

##### Color transformation matrix for later time-points

To allow for comparisons between simulated and real patterns at later time points, we define the transformation from the u,v,w to the color space through Principal Component Analysis (PCA) by finding the eigenvectors and eigenvalues of the covariance matrix defined as

$$Cov\left(X\right)=\frac{1}{n}{\left(X-\bar{X}\right)}^{T}\left(X-\bar{X}\right)$$

where n denotes the number of scales and, given that there are three RD variables (and three color variables), X is a n×3 data points matrix. For both color and u,v,w spaces, we can extract three orthogonal eigenvectors of which the one corresponding to the largest eigenvalue denotes the axis along which the data points exhibit the largest variation. Hence, the eigenvalues and eigenvectors form an ellipsoid approximately fitted to the data points: the ellipsoid center is the centroid of the data points; the ellipsoid axes and radii are the eigenvectors and the square root of eigenvalues, respectively. Hence, the transformation between the color and u,v,w spaces is defined as the matrix that maps the two ellipsoids from these two spaces. We then assume that *(i)* the eigenvectors of the color and RD variables are the columns of the matrices $V_{C}$, and $V_{u}$, respectively and *(ii)* the eigenvalues are the diagonal matrices $D_{C}$ and $D_{u}$, respectively. Hence, the affine transformation matrix from RD variable to color space reads

$$T_{e}\left(C,u\right)=\left(\begin{array}{cc} Q(C,u) & \bar{C}-Q(C,u)\bar{u}\\ 0 & 1 \end{array}\right)$$

in which $\bar{C}$ and $\bar{u}$ are the color and RD data points centroids, respectively, and Q(C,u) is the rotation and scaling matrix which reads

$$Q\left(C,u\right)=V_{C}D_{C}^{\frac{1}{2}}{\left(V_{u}D_{u}^{\frac{1}{2}}\right)}^{-1}$$

Then, the color of each scale is computed as

(Equation 31) $$\left(\begin{array}{c} C\\ 1 \end{array}\right)=T_{e}\left(C,u\right)\left(\begin{array}{c} u\\ 1 \end{array}\right)$$

#### Bayesian optimization of RD parameters

We optimize RD parameters using *Bayesian Optimization* (bayesopt library in MATLAB R2021a with parallel sampling), a machine-learning global minimization problem, defined as $\underset{x\in X}{\min\;}f\left(x\right)$, in which f(x) is the objective function. According to Frazier,^26^ this approach is efficient in minimising objective functions that are continuous, expensive to evaluate, have less than 20 dimensions and whose feasible set X is a hyper-rectangle or d-dimensional simplex.

##### Gaussian process regression

The core of the method is a random Gaussian process (*i.e.*, a generalization of the multivariate normal distribution to infinite dimensions) which enables sampling random functions. Here, we use this process to approximate the objective function. Let's assume that we already know a ‘training dataset’ of N outputs of the function f(x) as a vector f associated with N input vectors stored in the matrix X:

$$f=\left(\begin{array}{c} f(x_{1})\\ \vdots \\ f(x_{N}) \end{array}\right),X=\left(\begin{array}{c} x_{1}\\ \vdots \\ x_{N} \end{array}\right)$$

in which, $x_{i}\in R^{d}$ is a row vector containing optimisable variables. We know that the function f is sampled from a Gaussian process defined as

$$f\left(x\right)∼GP\left(m\left(x\right),k\left(x,x^{\prime }\right)\right)$$

in which m and k are mean and covariance functions, respectively. Now, we would like to estimate $f_{*}=f\left(x_{*}\right)$ at any arbitrary point $x_{*}$, *i.e.*, we look for $P\left(f_{*}|f\right)$, the probability of observing $f_{*}$ given the known output vectors f. By definition, any finite sets of data chosen from the Gaussian process exhibit a normal joint distribution. Since both $f_{*}$ and the output vector f are sampled from the same Gaussian process, they have a normal joint probability $P\left(f,f_{*}\right)$ which reads

$$\left(\begin{array}{c} f\\ f_{*} \end{array}\right)∼P\left(f,f_{*}\right)=N\left(\left(\begin{array}{c} m\\ m\left(x_{*}\right) \end{array}\right),\left(\begin{array}{cc} k & k_{*}^{T}\\ k_{*} & k\left(x_{*},x_{*}\right) \end{array}\right)\right)$$

in which m is the mean vector and $k,k_{*}$ are covariance block matrices defined as

$$m=\left(\begin{array}{c} m\left(x_{1}\right)\\ \vdots \\ m\left(x_{N}\right) \end{array}\right),\;k=\left(\begin{array}{ccc} k\left(x_{1},x_{1}\right) & \ldots & k\left(x_{1},x_{N}\right)\\ \vdots & \ddots & \vdots \\ k\left(x_{N},x_{1}\right) & \ldots & k\left(x_{N},x_{N}\right) \end{array}\right),\;k_{*}=\left(\begin{array}{c} k\left(x_{1},x_{*}\right)\\ \vdots \\ k\left(x_{N},x_{*}\right) \end{array}\right)$$

We can compute the conditional probability $P\left(f_{*}|f\right)$ as

$$P\left(f_{*}|f\right)=\frac{P\left(f_{*},f\right)}{P\left(f\right)}=\frac{P\left(f_{*},f\right)}{\int P\left(f_{*},f\right)df_{*}}$$

It can be shown that $P\left(f_{*}|f\right)$, from which $f_{*}$ can be sampled, is a normal distribution

$$f_{*}∼P\left(f_{*}|f\right)=N\left(m_{\mathrm{post}}\left(x_{*}\right),k_{\mathrm{post}}\left(x_{*},x_{*}^{'}\right)\right)$$

in which

$$m_{\mathrm{post}}\left(x_{*}\right)=m\left(x_{*}\right)+k_{*}^{T}k^{-1}\left(f-m\right)$$

$$k_{\mathrm{post}}\left(x_{*},x_{*}^{'}\right)=k\left(x_{*},x_{*}^{'}\right)-k_{*}^{T}k^{-1}k_{*}$$

The covariance function $k\left(x,x^{'}\right)$ has a crucial role on the performance of the Gaussian process and here we use:

(Equation 32) $$k\left(x,x^{'}\right)=\sigma_{f}^{2}\left(1+\sqrt{5}h+\frac{5}{3}h^{2}\right)\exp\left(-\sqrt{5}h\right)$$

in which

(Equation 33) $$h=\sqrt{\left(x-x^{\prime }\right)\sigma^{-2}{\left(x-x^{\prime }\right)}^{T}},\sigma =\left(\begin{array}{ccc} \sigma_{1} & 0 & 0\\ 0 & \ddots & 0\\ 0 & 0 & \sigma_{d} \end{array}\right)$$

The scalar $\sigma_{f}$ and the diagonal matrix σ are called *hyper-parameters* (that allow handling different length scales in parameter space) whose values are computed within the optimization loop. Here, we define the objective function as

(Equation 34) $$f=\frac{1}{n_{k}}\sum_{k}E_{k}^{\mathrm{pattern}}$$

where $n_{k}$ is the total number of observation time-points and $E_{k}^{\mathrm{pattern}}$ reads

$$\begin{array}{cc} E_{k}^{\mathrm{pattern}}=\frac{1}{n_{s}}\overset{7}{\sum_{i=0}} & |n_{k}^{\mathrm{sim}}\left(\mathrm{Green},i\right)-n_{k}^{\mathrm{obs}}\left(\mathrm{Green},i\right)|+\\ & \left|n_{k}^{\mathrm{sim}}\left(\mathrm{Black},i\right)-n_{k}^{\mathrm{obs}}\left(\mathrm{Black},i\right)\right| \end{array}$$

where $n_{s}$ is the total number of scales. The definition of nearest-neighbor statistics n(S,i) is given above in the section stochastic cellular automaton.

Note that the sensitivity analysis over the parameters of the RD model (Figure 7E) was performed by running, in each species, 5,000 simulations with perturbed (unoptimal) parameter values sampled from a uniform random distribution within the range ± $0.1\sigma_{1,\ldots ,d}$, where $\sigma_{1,\ldots ,d}$ are the hyper-parameters of the covariance function (Equation 33).

##### Gaussian process optimization loop

Starting with an initial parameter vector $x_{0}$, evaluation of the objective function $f\left(x_{0}\right)$ is iteratively accomplished through the following steps: *(i)* Generate initial condition $u\left(\tau_{0}\right)$ by converting the observed colors $C\left(\tau_{0}\right)$ and using the transformation defined in Equation 30; *(ii)* Solve the discrete-RD equations (Equation 20) for all time points ranging from $\tau_{0}$ to $\tau_{k}$; *(iii)* Convert the simulation results $u\left(\tau_{k}\right)$ to the Lab coordinate colors using the observed colors $C\left(\tau_{k}\right)$ and the transformation defined in Equation 31 and *(iv)* Evaluate the objective function f according to Equation 34. After point (iv), the output vector and input matrix are updated by adding the corresponding values to the end of f and X, respectively. We assume that f(x) can be drawn from the Gaussian process in which m(x)=0 and $k\left(x,x^{\prime }\right)$ is the covariance function given in Equation 32. We can use f and X to obtain the mean $m_{\mathrm{post}}$ and covariance $k_{\mathrm{post}}$ at any point in the parameter space. The latter two are used to guess the next sampling point. For that purpose, we define the improvement function as:

$$I\left(x_{*}\right)=\{\begin{array}{cc} f_{\min}-f_{*} & f_{\min}>f_{*}\\ 0 & f_{\min}\leq f_{*} \end{array}$$

in which $f_{\min}$ is the minimum values of the objective function evaluated so far. The improvement function simply indicates the amount by which the objective function is reduced with respect to the best value evaluated so far. For region in which the objective function exceeds $f_{\min}$, an improvement value of zero is assigned. The expectation of $I\left(f_{*}\right)$ has the following closed form^27^

(Equation 35) $$\;\begin{array}{ccc} E\left[I\left(x_{{}_{*}}\right)\right]=\int_{-\infty }^{\infty }I\left(x_{{}_{*}}\right)P\left(f_{*}|f\right)df_{*} & = & \left(f_{\min}-m_{\mathrm{post}}\left(x_{{}_{*}}\right)\right)\Phi \left(\frac{f_{\min}-m_{\mathrm{post}}\left(x_{{}_{*}}\right)}{k_{\mathrm{post}}\left(x_{{}_{*}},x_{{}_{*}}\right)}\right)+\\ & & k_{\mathrm{post}}\left(x_{{}_{*}},x_{{}_{*}}\right)\varphi \left(\frac{f_{\min}-m_{\mathrm{post}}\left(x_{{}_{*}}\right)}{k_{\mathrm{post}}\left(x_{{}_{*}},x_{{}_{*}}\right)}\right) \end{array}$$

where φ and Φ are the probability density function and cumulative density function of the standard normal distribution, respectively. Note that this function is much cheaper to evaluate than the objective function. The point to sample in the next iteration is the one that minimizes the expected improvement function in Equation 35. This classical minimization problem is efficiently handled by a gradient descent algorithm. The optimization loop is repeated until 1000 successive iterations have not generated any improvement.

##### Narrowing parameter space

Even-though the dRD solver is computationally very efficient, running the optimization loop over the full space of parameters

$$S_{full}=\left\{\left[c_{u},c_{v},c_{w},c_{1},\ldots ,c_{9},D_{u},D_{v},D_{w},P,q,r\right]:c_{u},c_{v},c_{w},c_{1},\ldots ,c_{9}\in R,D_{u},D_{v},D_{w},P,q,r\in R^{+}\right\}$$

is not feasible. To identify a suitable subset of parameters $S\subset S_{\mathrm{full}}$ on which to perform optimisation, we first take into account the redundancy in the dRD formulation. Indeed, the parameter P can be removed from the optimization loop as it is a multiplier of the diffusion coefficients $D_{u},D_{v},D_{w}$. Furthermore, we remove $D_{v}$ as $D_{u}=D_{v}$. Second, we use the 2-D continuous RD formulation to study the effect of varying pairs of decay and reaction coefficients $c_{u},c_{v},c_{w},c_{1},\ldots ,c_{9}$ on pattern generation. The corresponding 55 simulations are displayed as gradient plots (Figure S5) in which two coefficients are varying in space. For example, in the simulation labeled $c_{i}-c_{j}$, the variation of coefficients of variation is defined as

$$\begin{array}{c} c_{i}\left(x\right)=c_{i}+\left(\frac{\left|c_{i}\right|}{x_{\max}-x_{\min}}\right)\left(x-\frac{\left(x_{\min}+x_{\max}\right)}{2}\right)\\ c_{j}\left(y\right)=c_{j}+\left(\frac{\left|c_{j}\right|}{y_{\max}-x_{\min}}\right)\left(y-\frac{\left(y_{\min}+y_{\max}\right)}{2}\right) \end{array}$$

where $x_{\min},x_{\max},y_{\min}$ and $y_{\max}$ are the domain limits and, $c_{i}$ and $c_{j}$ are the coefficient values proposed in Manukyan et al.^12^, *i.e.*,

$$\begin{array}{ccccc} c_{u}=0.020 & , & c_{v}=0.025 & , & c_{w}=0.06,\\ c_{1}=-0.04 & , & c_{2}=-0.056 & , & c_{3}=0.382,\\ c_{4}=-0.05 & , & c_{5}=0.0 & , & c_{6}=0.25,\\ c_{7}=0.016 & , & c_{8}=-0.03 & , & c_{9}=0.24 \end{array}$$

To produce these plots, we neglect domain growth and set the other coefficients to the values proposed in Manukyan et al.^12^, *i.e.*,

$$D_{u}=D_{v}=1.125,\;D_{w}=12.5,\;P=0.00889$$

All simulations start from the HSS, perturbed with small random fluctuations, and are run to steady-state. In most of the gradient plots, a similar diversity of patterns appears, suggesting that one might optimize the model with only a subset of parameters. To test this conjecture, we set up optimization experiments in which parameters are added iteratively to S. First, we restrict S to q and r and run the optimisation loop (all other parameters are set to the values proposed in Manukyan et al.^12^). Then, we repeat the optimization loop for all possible subsets corresponding to the inclusion of one additional parameter in S. The parameter whose addition generates the lowest value of the objective function is permanently added to S. We repeat this iterative process of adding the next best parameter until no reduction of the objective function is observed. Table S1 shows the objective function values obtained during the iterative addition process performed for TL1: the columns indicate the number of parameters in S, and the rows are ordered by parameters iteratively identified as most useful (because exhibiting the lowest value, shown in bold, of the objective function). Table S1 indicates that the optimal choice of subset S for TL1 is

$$S=\left\{\left[c_{v},D_{u},D_{w},q,r\right]:c_{v}\in R,D_{u},D_{w},q,r\in R^{+}\right\}$$

Note that we perform this identification of best subset S independently for each individual of each species. After performing Bayesian optimisation (again, independently for each individual of each species, see above) on this parameter vector, we obtain the optimized dRD model parameters listed in Table S2. Note that an optimal subset S was found for all individuals of all species after optimising only 3 to 5 parameters (all other parameters did not require modifying their values from Manukyan et al.^12^).

#### Uncertainty in skin thickness spatial distribution and production of 3D super-Gaussian heterogeneous networks

Although the acquisition of skin-scale surface micro-geometry captures the continuous variation of skin height, it does not capture the variation of the bottom boundary of the domain. In other words, we assume that the thinning of the skin at scale borders is the same at all borders of all scales, hence, the use of a single parameter P for the reduction of RD diffusion coefficients at all one-dimensional edges^12^ in the 2D-cRD model. To quantify how much real skin geometry deviates from this assumption, we perform 3D geometry reconstruction of a patch of dorsal skin for each of the five species. For example, the patch for ocellated lizard comprises 24 scales (10 green and 14 black) and was reconstructed using 707 images (4082 × 3072 pixels each with 1 pixel = 1.2 μm) acquired with high-resolution episcopic microscopy (HREM^14^^,^^23^). Sections generated by HREM are intrinsically-aligned, making the volume rendering straightforward. We then perform image analyses^14^ to identify the top and bottom boundaries of the skin as well as the positions occupied by melanophores. The former allows defining the full skin 3D domain whereas the latter provides an estimate of the restricted 3D domain populated by chromatophores. Indeed, as RD components are associated to chromatophores themselves, it is likely that relevant statistics on skin thickness apply to the restricted domain rather than to the whole skin depth.

The HREM data allows us to extract, for a dorsal patch of skin in each species, the spatial distributions of skin thickness (d) and of thickness $d_{m}$ in the domain restricted to chromatophores. We then compute the mean distance S among neighboring scales at their highest point, as well as the means among the top-surface highest point of scales (${\bar{h}}_{c}$) and among the heights of edges (${\bar{h}}_{e}$). We then construct a ‘noisy’ 3D lattice (an example is shown in Figure 5B for *T. lepidus*) in which heights $h_{c}$ and $h_{e}$ (*i.e.*, at the centers and at the edges of the super-Gaussian scales, respectively) vary with standard deviations $\sigma_{c}$ and $\sigma_{e}$. Starting from the HREM-observed average values of S, ${\bar{h}}_{c}$ and ${\bar{h}}_{e}$, we optimise ${\bar{h}}_{c}$, ${\bar{h}}_{e}$, p, σ, $\sigma_{c}$ and $\sigma_{e}$ to obtain a depth-map histogram of the noisy lattice (*e.g.*, Figure 5C for *T. lepidus*), hence, a skin thickness distribution, highly similar to that observed in the HREM data. We then construct 1,000 domains of 64 super-Gaussian scales, represented as 3D curvilinear structured grids, by keeping S and p constant, but sampling $h_{c}$ (for each bump) and $h_{e}$ (for each edge) from the Normal distributions $N\left({\bar{h}}_{c},\sigma_{c}\right)$ and $N\left({\bar{h}}_{e},\sigma_{e}\right)$ with optimized parameters. Finally, using the mapping $d_{m}\left(d\right)$ discussed above, we compute the bottom boundary of each domain by subtracting $d_{m}$ from the top boundary, hence, producing models of the skin domain restricted to chromatophores (turquoise volume illustrated in Figure 5B for *T. lepidus*). HREM data acquisition, optimization of parameters ${\bar{h}}_{c}$, ${\bar{h}}_{e}$, p, σ, $\sigma_{c}$, as well as sampling of $h_{c}$ and $h_{e}$ from the Normal distributions $N\left({\bar{h}}_{c},\sigma_{c}\right)$ and $N\left({\bar{h}}_{e},\sigma_{e}\right)$, are performed independently for each species. We also generate for each species a reference hexagonal 3D lattice of identical super-Gaussian bumps, with super-Gaussian parameters σ and p, and with the centers of all bumps and all the edges set to optimized ${\bar{h}}_{c}$ and ${\bar{h}}_{e}$, respectively.

#### Color measurement uncertainty and Lyapunov spectrum analysis

One powerful approach to estimate the degree of sensitivity of a dynamical system is to compute how fast two trajectories diverge when they initially differ by an arbitrary small difference. To this aim, we first define $C^{\mathrm{ref}}=\left(\begin{array}{c} C_{1},\ldots ,C_{n_{s}} \end{array}\right)$ as the reference $3\times n_{s}$ matrix containing the states (in CIELAB colors) of all scales of the observed juvenile lizard. We then generate 2,000 ‘noisy initial conditions’ by adding random noise to the reference matrix, *i.e.*, $C=C^{\mathrm{ref}}+sC^{'}$, where the scalar s and the matrix $C^{\prime }$ are randomly sampled from the uniform and 3-variate normal distributions, U(0,1) and $N\left(0,\Sigma_{\circ }^{\mathrm{ref}}\right)$, respectively. Note that $\Sigma_{\circ }^{\mathrm{ref}}$ is the covariance matrix of $C^{\mathrm{ref}}$. We then use a color transformation matrix (see STAR Methods, section Juvenile initial condition (IC) and color transformation matrix) to transfer each of these 2,000 noisy initial conditions to the space of RD variables for performing numerical simulations. For each initial condition, the state of the system at time τ is defined as:

$$S_{\tau }=\left[u_{1}\left(\tau \right),v_{1}\left(\tau \right),w_{1}\left(\tau \right),...,u_{n_{s}}\left(\tau \right),v_{n_{s}}\left(\tau \right),w_{n_{s}}\left(\tau \right)\right]$$

and we study its time evolution as a trajectory in the $R^{3\times n_{s}}$ phase space parametrized by the time variable $\tau_{\circ }\leq \tau \leq \tau_{f}$, where $\tau_{\circ }$ and $\tau_{f}$ are the juvenile (initial condition) and final (adult) time points, respectively. We then measure, at each time τ, the Euclidean distance $\delta_{\tau }=∥S_{\tau }-S_{\tau }^{\mathrm{ref}}∥$ between the state of the system ($S_{\tau }$) and the state ($S_{\tau }^{\mathrm{ref}}$) of the reference (*i.e.*, the latter is the trajectory starting from the observed unperturbed juvenile colors). We then use the Lyapunov exponent λ as an estimate of the rate of divergence between the two trajectories:

$$\lambda =\lim_{\tau \to \infty }\;\lim_{\delta_{\circ }\to 0}\frac{1}{\tau -\tau_{\circ }}\;\log\frac{\delta_{\tau }}{\delta_{\circ }}$$

where $\delta_{\circ }=\delta_{\tau_{\circ }}$. Figure 6A shows, for each of the 2,000 simulations, the trajectory of $\log\left(\delta_{\tau }/\delta_{\circ }\right)$ as a function of time. The Lyapunov exponent is then simply derived as the slope of the curve (computed with least-square fit) for early time points, *i.e.*, before saturation of $\delta_{\tau }$. The positive value of the mean (± SD) Lyapunov exponent (λ=0.0287±0.0004 for TL1) confirms that the system is unstable.

### Quantification and statistical analysis

All statistical analyses were performed with MATLAB R2021a. Statistical details can be found in the figure legends, results section and the STAR Methods.

## Data Availability

All data needed to evaluate the conclusions in the paper are present in the paper and/or the supplemental information. Executables implementing the 2D and 3D RD processes are available at https://github.com/LANEvol/Discrete-RD.git and https://github.com/LANEvol/RD-Curvilinear.git, respectively, for repeating the numerical simulations presented here.

## Supplemental information

Document S1. Figures S1–S7 and Tables S1–S4

Data S1. 16D nearest-neighbor error vectors (ocellated lizard TL1) projected in PC1-PC2-PC3 space, related to Figure 2 Interactive 3D graph (xhtml format for viewing in web browsers) plotting all error vectors for sCA, Lenz-Ising and RD simulations (cf. main Figure 2A).

Data S2. 16D nearest-neighbor error vectors (ocellated lizard TL2) projected in PC1-PC2-PC3 space, related to Figure 2 Interactive 3D graph (xhtml format for viewing in web browsers) plotting all error vectors for sCA, Lenz-Ising and RD simulations (cf. Figure S2C).

Document S2. Article plus supplemental information

Video abstract
