# Supplementary material for: Modeling convergent scale-by-scale skin color patterning in multiple species of lizards
Source: Curr Biol. 2022 Dec 5;32(23):5069–5082.e13. doi: 10.1016/j.cub.2022.10.044 (PMC9763091; doi:10.1016/j.cub.2022.10.044)
Supplement: Document S1. Figures S1–S7 and Tables S1–S4 [file mmc1.pdf]

Current Biology, Volume *32*

Supplemental Information

Modeling convergent scale-by-scale skin color patterning in multiple species of lizards

Ebrahim Jahanbakhsh and Michel C. Milinkovitch

Current Biology, Volume *32*

Supplemental Information

Modeling convergent scale-by-scale skin color patterning in multiple species of lizards

Ebrahim Jahanbakhsh and Michel C. Milinkovitch

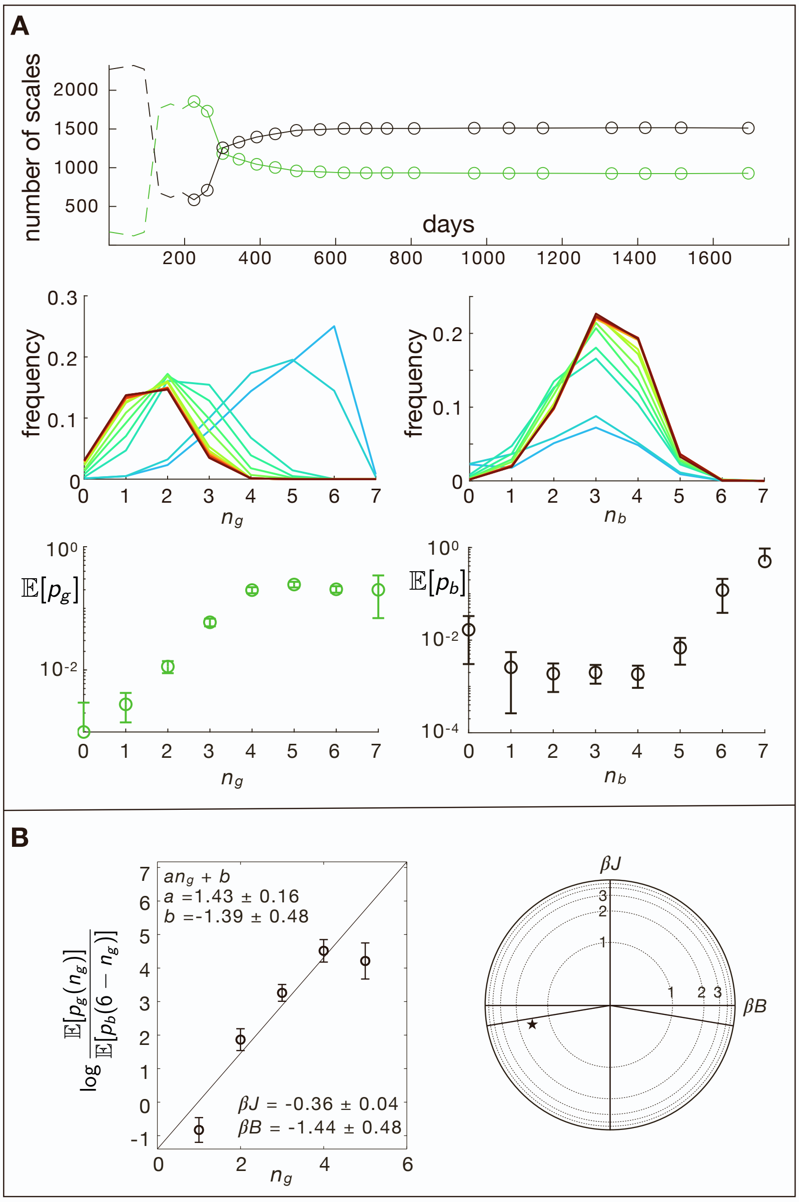

Figure S1 | Neighbourhood statistics and stochastic models for ocellated lizard; related to Figure 2 — (**A**) Top panel: time history of the number of green (green circles) and black (black circles) scales observed in ocellated lizard individual TL1; scale colours are thresholded to green or black by applying K-mean clustering. Middle panel: relative frequencies of green (left) and black (scales) as a function of $n_{\mathrm{g/b}}$, *i.e.*, the number of isochromatic direct neighbours; lines of different colours correspond to different observed time-points (*i.e.*, circles in top panel), from blue (juvenile state) to red (adult). Lower panel: expected probability of colour change ($\mathbb{E}[p_{\mathrm{g/b}}]$) for green/black scales as a function of $n_{\mathrm{g/b}}$ (circles); error bars indicate the 0.95% confidence interval corresponding to the Binomial distribution. (**B**) Left panel: mean values (±SD) of $\log(\mathbb{E}[p_g(n_g)]/(\mathbb{E}[p_b(6-n_g)])$ (using the data from (A)) with the best affine fit ($an+b$) and corresponding Lenz-Ising model parameters βB and βJ (non-Glauber dynamics[14]). Right panel: optimised Lenz-Ising model parameters (black star) at finite temperature, for individual TL1, plotted in polar coordinates with radial coordinate mapped to a finite range. Oblique solid lines indicate the separation between ferromagnetic and anti-ferromagnetic regions in the triangular Lenz-Ising model.

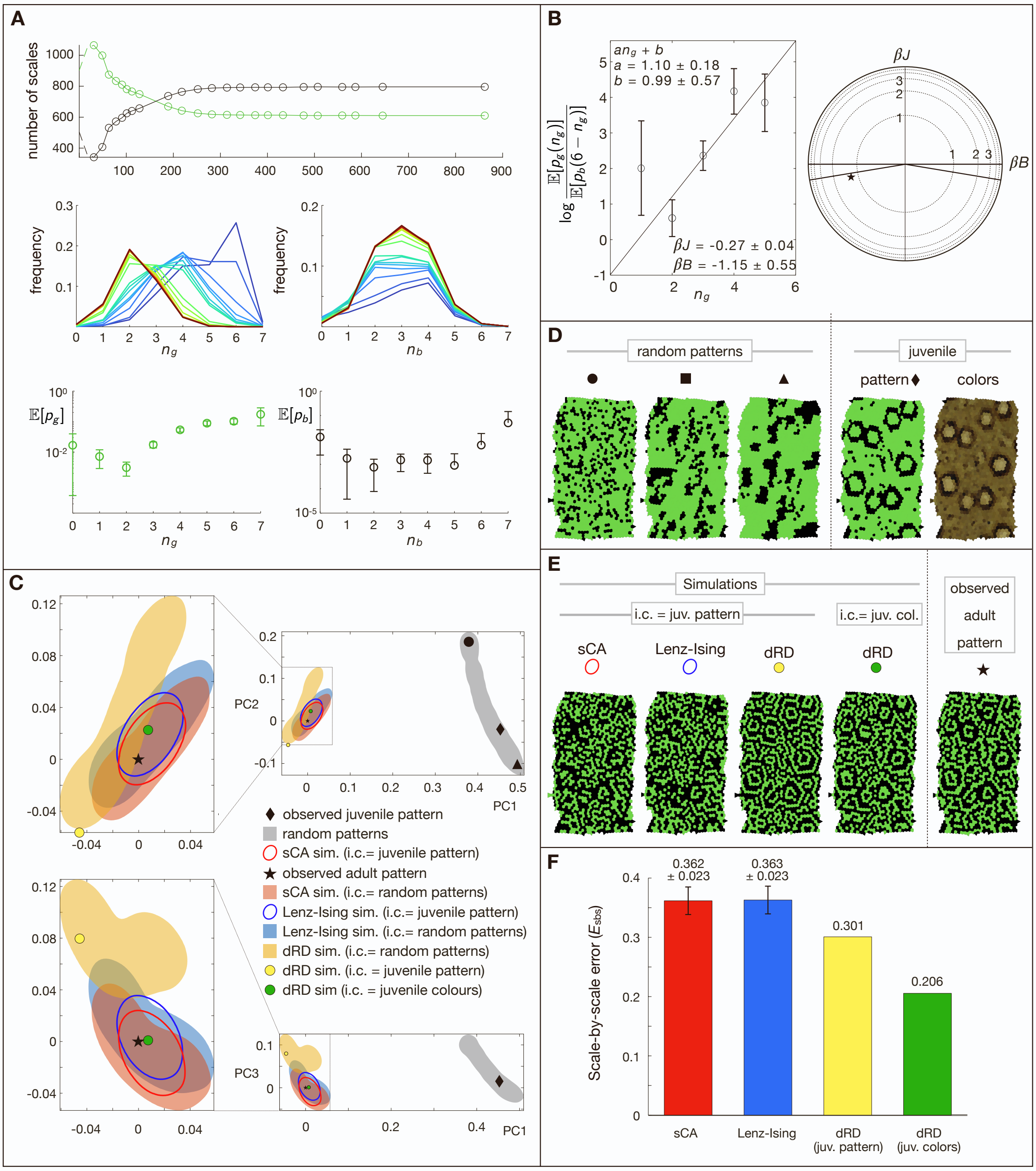

Figure S2 | Neighbourhood statistics and their predictability in ocellated lizard individual TL2; related to Figures 2 and 4 — (**A**) Same analyses as in figure S1A but for individual ocellated lizard TL2. (**B**) Same analyses as in figure S1B but for individual ocellated lizard TL2. (**C**) Same analyses as in main Figure 2 but for individual ocellated lizard TL2. (**D**) Different initial conditions used for simulations; their localisation in PC1-PC2-PC3 space are shown with the corresponding geometrical symbols. (**E**) Adult patterns simulated with different initial conditions (i.c.): juvenile (juv.) pattern (= scale colours thresholded to green or black) and juvenile colours (col.) are both shown in D. (**F**) Histogram comparing scale-by-scale errors (mean ± SD) of adult patterns simulated with different models.

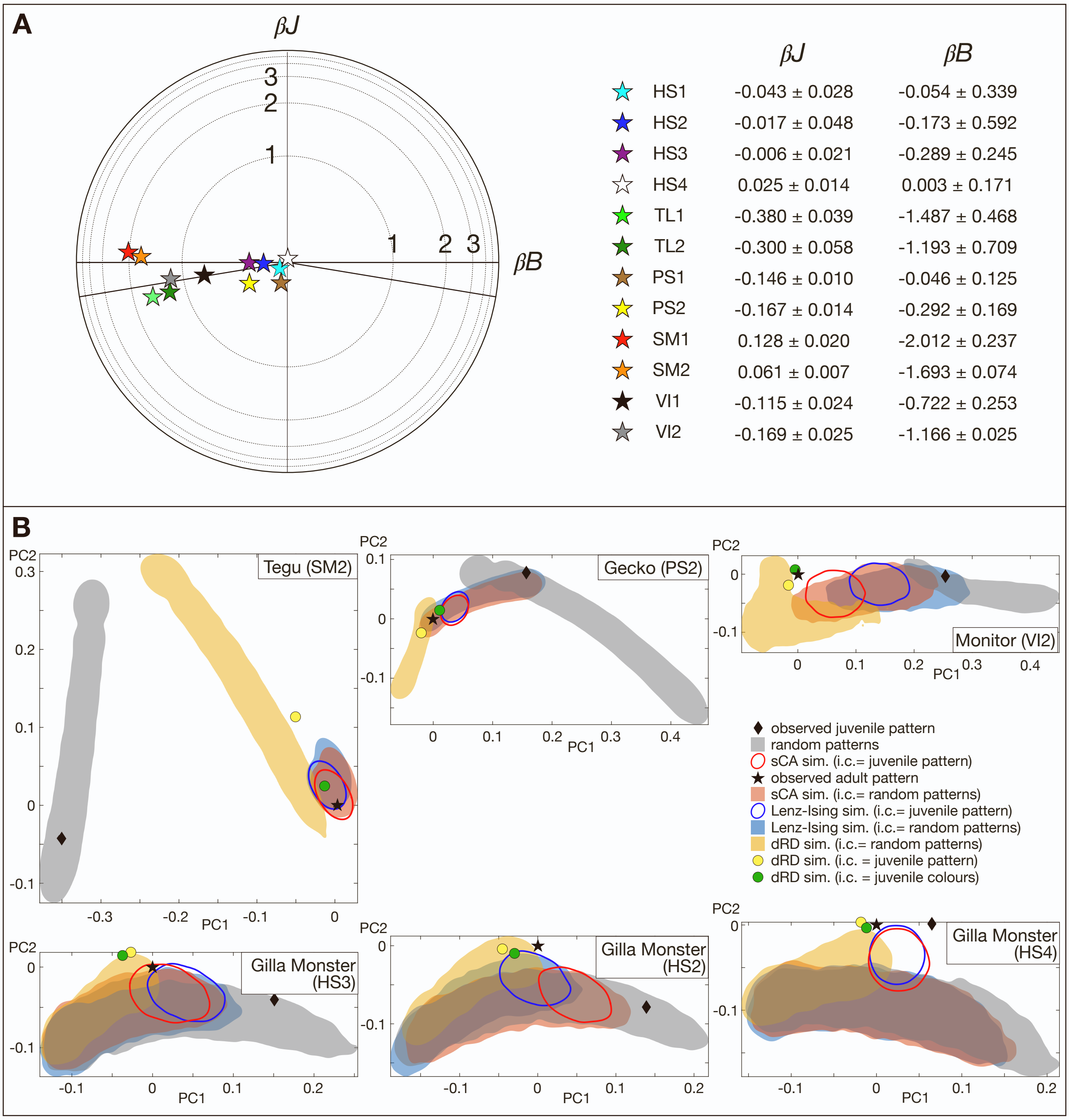

Figure S3 | Lenz-Ising parameters and prediction of neighbourhood statistics in four other species; related to Figure 3 — (A) Optimised Lenz-Ising model parameters (stars) for all individuals of all species plotted in polar coordinates with radial coordinate mapped to a finite range. Oblique solid lines indicate the separation between ferromagnetic and anti-ferromagnetic regions in the triangular Lenz-Ising model. (**B**) Projections on the PC1-PC2 plane of the 16D nearest-neighbour error vectors (in comparison to the corresponding observed adult pattern, black stars) of patterns simulated with sCA (red ellipse and red shading), Lenz-Ising (blue), and dRD (yellow) models. Red ellipses, blue ellipses and yellow spots show adult patterns simulated from the corresponding observed juvenile patterns (black diamonds), whereas red, blue and yellow shadings show adult patterns simulated from random patterns (grey areas) as initial condition. The green spots show the adult dRD-patterns simulated from the juvenile colours in the corresponding individual. Ellipses and border of shadings indicate 1% density isolines.

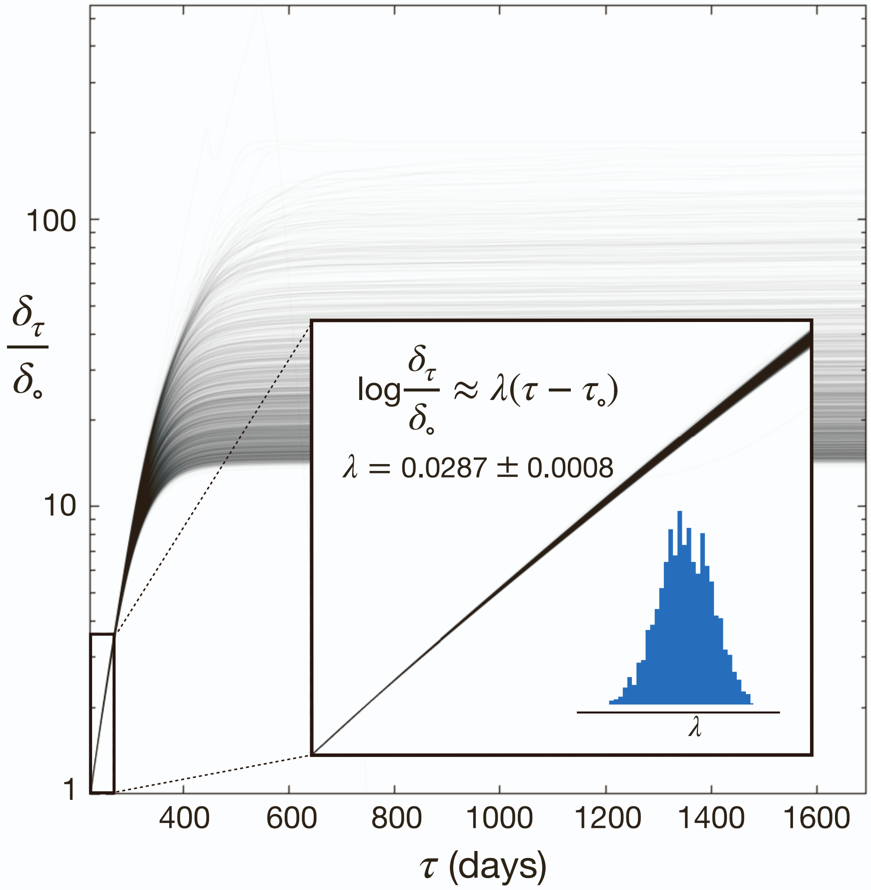

Figure S4 | Lyapunov spectrum analysis and colour measurement uncertainty in ocellated lizard individual TL1; related to Figure 6 and Data S1B — Time-evolution trajectories of 2,000 'perturbed' simulations, *i.e.*, started at random small colour differences (E°_{sbs}) from the observed juvenile colours. E°_{sbs} is then translated to the Euclidean distances ($\delta_\circ$) in RD space; δ_τ = distance at time τ between the state of each perturbed simulation and the state of the reference trajectory (starting from observed juvenile state); (Data S1B). The positive value of the mean (± SD) Lyapunov exponent ($\lambda =$ slope of $\log(\delta_\tau/\delta_\circ)$ for early time points) confirms the instability of the system. The blue histogram in the inset indicates the distribution of λ across all simulations.

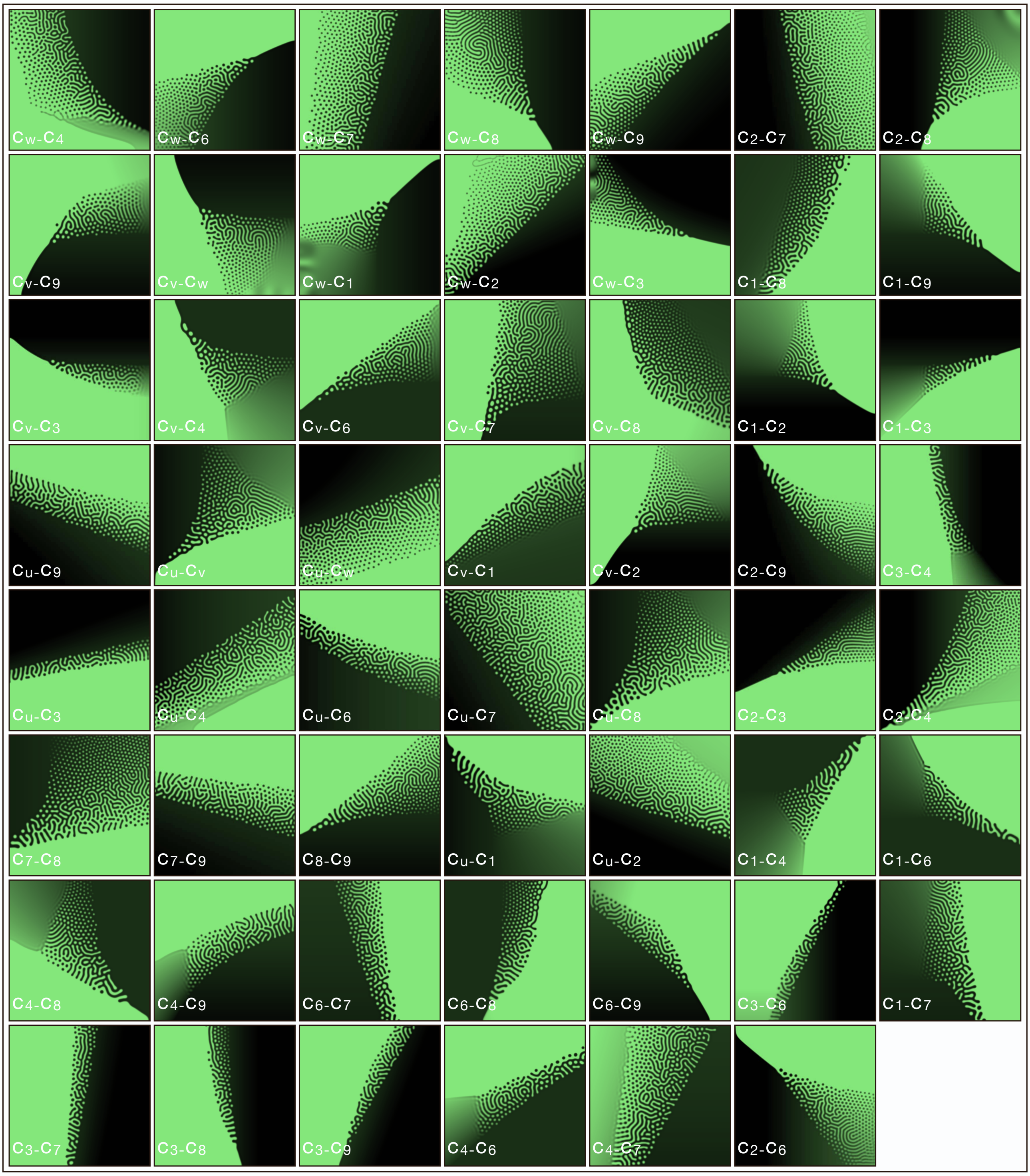

Figure S5 | Pairwise parameter gradient plots; related to Figure 4 and STAR methods — Different patterns generated by linear variation of RD model parameters. First and second varying parameters (white font) correspond to the horizontal and vertical directions, respectively. The limit of variations in each direction is $\pm 50\,\%$ of the absolute values given in Eq. (35).

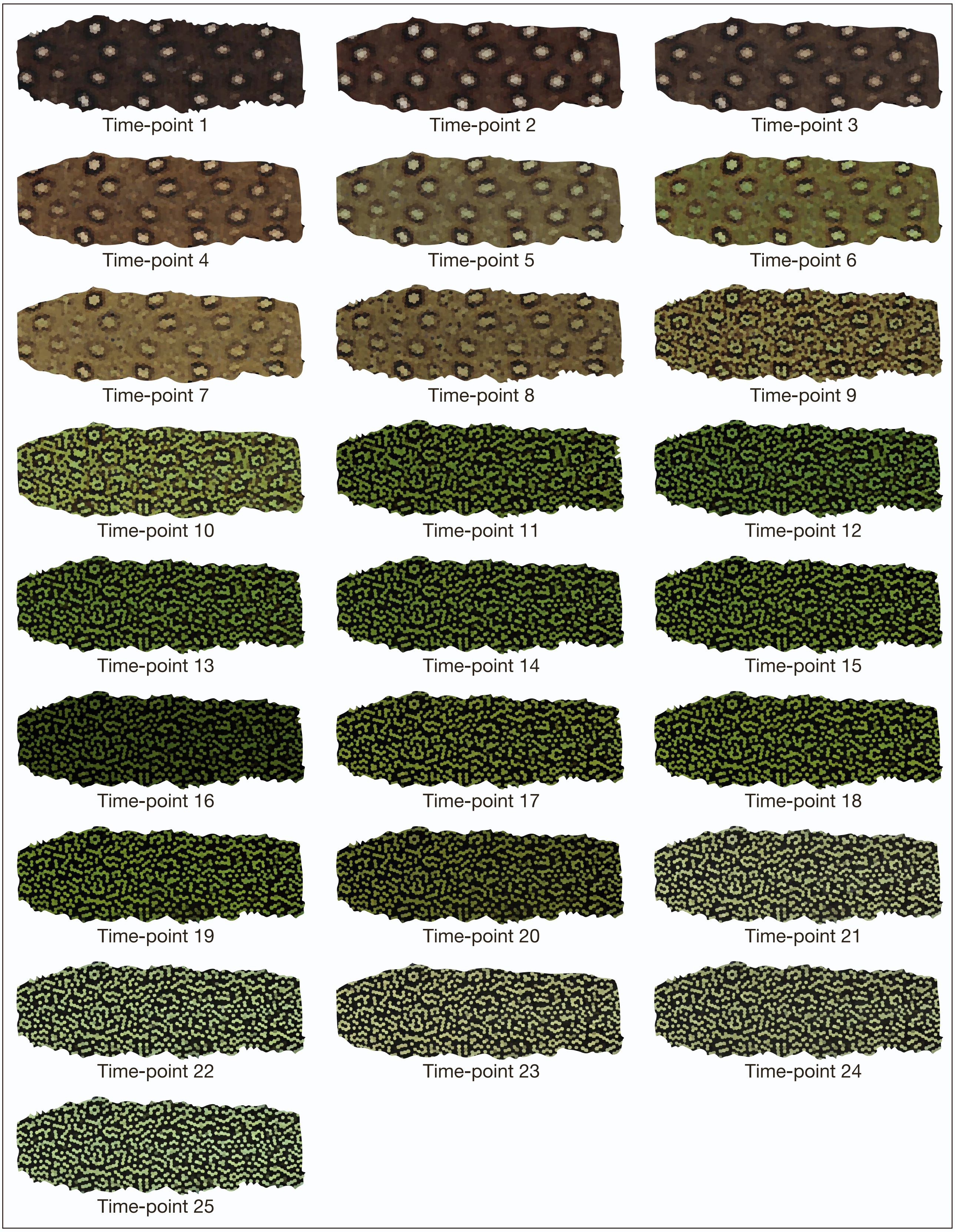

Figure S6 | Time evolution of observed scale colours of ocellated lizard; related to Figure 1B-D and STAR methods — Scale colours at 25 time-points for the patch of skin analysed in ocellated lizard TL1 (cf. Figure 1B). The colour of each polygonal scale is set to the mean albedo of its pixels at the corresponding time-point (see STAR methods).

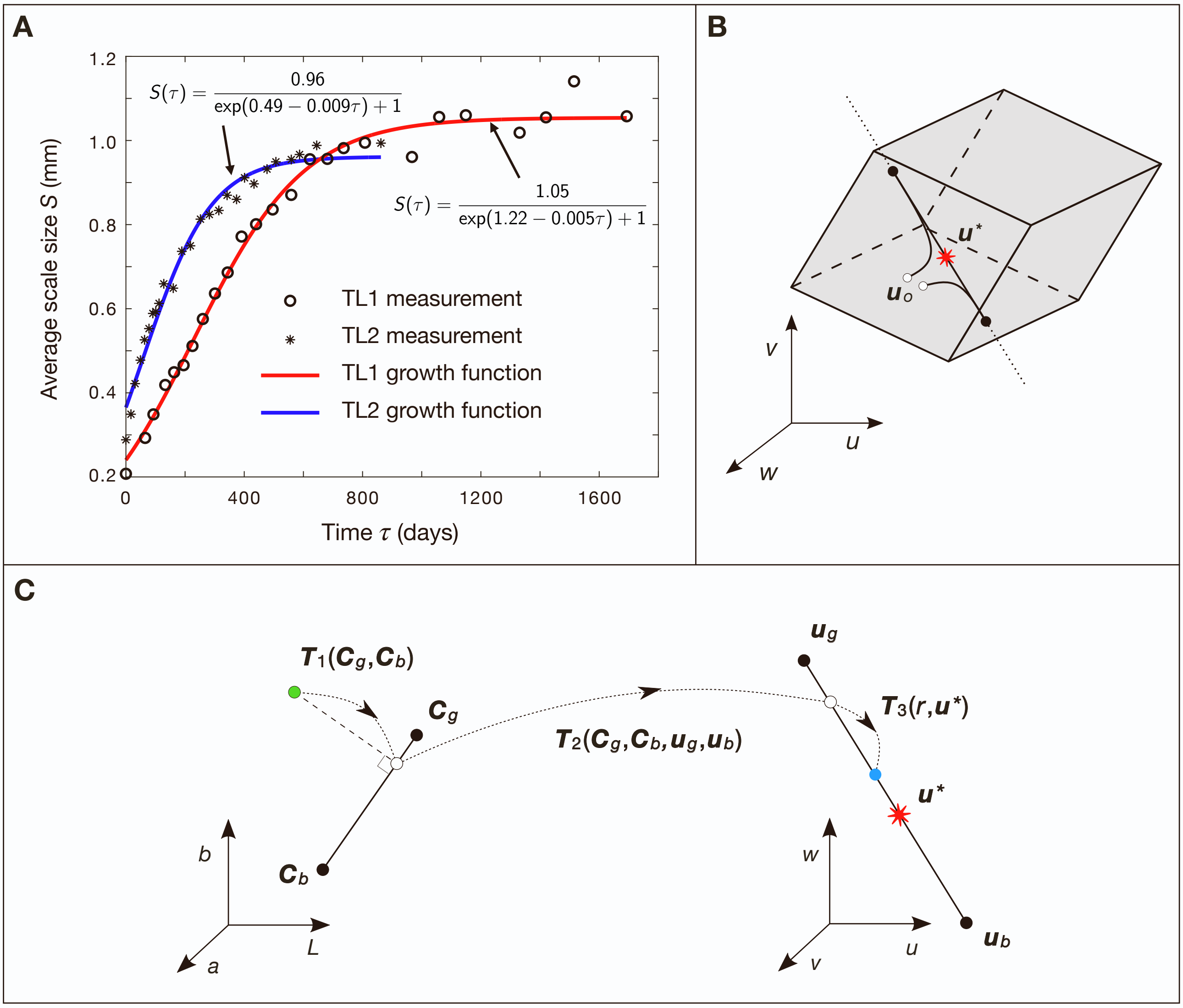

Figure S7 | Growth model and colour transformation to RD variables; related to STAR methods — (**A**) The time history of mean scale size (among edge lengths of all scales) for ocellated lizards TL1 and TL2. Solid lines show the best fitted logistic functions. (**B**) Schematic trajectories of scales in u, v, w space shown as curved lines; trajectories start from $\boldsymbol{u}_{\circ}$ (white circles) close to the HSS (red star) and approach the dominating eigenvector line (Eq. (29)) shown as a solid black line which extremities (black circles) intersect the grey planes described by Eq. (24). (**C**) Transformation between CIELAB colour space and u, v, w space using matrices $\boldsymbol{T}_1$, $\boldsymbol{T}_2$, and $\boldsymbol{T}_3$; the green dot indicates, for a given scale, its observed juvenile colour and the blue dot, located on the $\boldsymbol{u}_g - \boldsymbol{u}_b$ line (*i.e.*, the dominating eigenvector line), indicates the corresponding initial RD component values used for simulations.

dimensions =	2	3	4	5	6
q, r	**0.2448**	–	–	–	–
c_v	–	**0.1774**	–	–	–
$D_{u,v}$	–	0.2373	**0.1167**	–	–
D_w	–	0.2308	0.1774	**0.1086**	–
c_u	–	0.2257	0.1774	0.1103	0.1086
c_w	–	0.2246	0.1774	0.1143	0.1086
c_1	–	0.1809	0.1774	0.1167	0.1086
c_2	–	0.2268	0.1774	0.1167	0.1086
c_3	–	0.2129	0.1774	0.1167	0.1086
c_4	–	0.2247	0.1774	0.1167	0.1086
c_5	–	0.2264	0.1774	0.1167	0.1086
c_6	–	0.2123	0.1774	0.1167	0.1086
c_7	–	0.2252	0.1774	0.1167	0.1086
c_8	–	0.1812	0.1774	0.1167	0.1086
c_9	–	0.2121	0.1774	0.1167	0.1086

Table S1 | Optimised values, for individual TL1, of the objective function f (equation 34 in STAR methods) during iterative addition of different parameters (left column) to $\mathbb{S}$; related to STAR methods — We start by optimising 2 free parameters (q and r), while keeping all others at the values of ref. 12 in the main text, and obtain an f value of 0.2448. We then make all combinations of q, r and i (column 3), where i is each of the remaining 14 reaction, decay, and diffusion parameters. For each i, we optimise q, r and i, and select the parameter i that gives the best value of f (here, 0.1774 for c_v *vs.* higher values for all other parameters). We then make all combinations of q, r, c_v and i, where i is each of the remaining 13 parameters (column 4). The procedure is iterated until no improvement in the value of function f is obtained. In this example, (individual TL1), the optimal value is obtained after optimising 5 parameters highlighted by shaded cells.

	q [-]	r [-]	c_u [d^{-1}]	c_v [d^{-1}]	c_w [d^{-1}]	$D_{u,v}$ [d^{-1} l^2]	D_w [d^{-1} l^2]	c_1 [d^{-1}]	c_2 [d^{-1}]	c_3 [z d^{-1}]	c_4 [d^{-1}]	c_5 [d^{-1}]	c_6 [z d^{-1}]	c_7 [d^{-1}]	c_8 [d^{-1}]	c_9 [z d^{-1}]
**TL1**	**2.9**	**0.23**	*0.02*	**0.03**	*0.06*	**0.07**	**21.8**	-0.04	-0.056	0.382	-0.05	0	0.25	0.016	-0.03	0.24
**TL2**	**2.25**	**0.33**	*0.02*	*0.025*	*0.06*	**0.33**	**22.9**	-0.04	-0.056	0.382	-0.05	0	0.25	0.016	-0.03	**0.239**
**PS1**	**3.15**	**0.63**	0.02	**0.022**	0.06	1.125	12.5	-0.04	-0.056	**0.384**	-0.05	0	0.25	0.016	-0.03	0.24
**PS2**	**4.9**	**0.85**	0.02	0.025	0.06	1.125	**21.5**	-0.04	-0.056	0.382	-0.05	0	0.25	**0.015**	-0.03	0.24
**SM1**	**4.8**	**0.95**	0.02	0.025	0.06	**0.055**	12.5	-0.04	-0.056	0.382	-0.05	0	**0.212**	0.016	-0.03	0.24
**SM2**	**1.04**	**0.93**	0.02	0.025	0.06	**0.06**	12.5	-0.04	-0.056	0.382	-0.05	0	**0.222**	0.016	-0.03	0.24
**HS1**	**1.9**	**0.07**	0.02	0.025	0.06	**0.27**	12.5	-0.04	-0.056	0.382	-0.05	0	**0.238**	0.016	-0.03	0.24
**HS2**	**1.7**	**0.23**	**0.018**	0.025	0.06	**0.43**	12.5	-0.04	-0.056	0.382	-0.05	0	0.25	0.016	-0.03	0.24
**HS3**	**2.5**	**0.16**	0.02	0.025	0.06	**0.21**	12.5	-0.04	-0.056	0.382	-0.05	0	**0.238**	0.016	-0.03	0.24
**HS4**	**1.13**	**0.23**	0.02	0.025	0.06	**0.27**	12.5	-0.04	-0.056	0.382	-0.05	0	0.25	0.016	-0.03	0.24
**VI1**	**1.1**	**0.05**	0.02	0.025	0.06	**0.11**	12.5	-0.04	-0.056	0.382	**-0.053**	0	0.25	0.016	**-0.039**	0.24
**VI2**	**2.7**	**0.61**	0.02	0.025	0.06	**0.43**	12.5	**-0.037**	-0.056	0.382	-0.05	0	**0.228**	0.016	-0.03	0.24

Table S2 | RD parameters for all individuals; related to STAR methods — Shaded cells indicate values optimised using Bayesian machine-learning following the procedure illustrated in table S1. Other values are from ref. 12 in the main text. Units are indicated between brackets ([-] = dimensionless, d = day, $l \approx 86\,\mu m$ and z = dimensions of the corresponding *u, v* and *w* RD variables).

		Model parameters		
		SM1	SM2	VI1
Initial condition	SM1	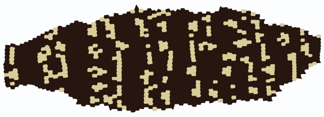 **(0.037)**	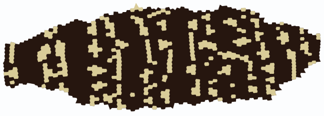 **(0.065)**	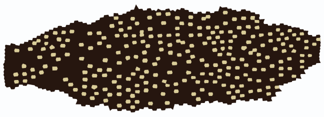 **(0.296)**
	SM2	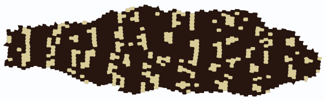 **(0.070)**	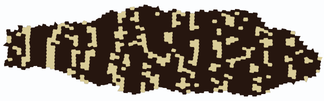 **(0.056)**	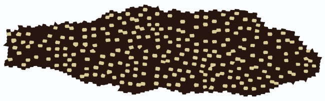 **(0.281)**
	VI1	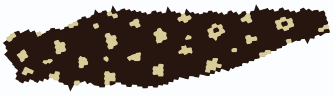 **(0.407)**	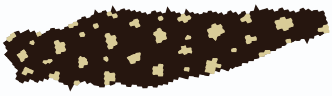 **(0.380)**	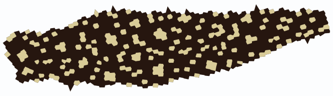 **(0.077)**

Table S3 | Exchanging sets of RD parameters among individuals within and between species (black and white tegu *versus* mangrove monitor lizard); related to Figure 3 — Colour patterns of two tegus ($E_{16\mathrm{D}}$= 0.081 for SM1 *versus* SM2) and one monitor lizard (VI1) simulated after exchanging their optimised RD parameters. Numbers in parentheses indicate $E_{16\mathrm{D}}$ errors when compared to the observed adult pattern.

		Model parameters		
		TL1	TL2	SM1
Initial condition	TL1	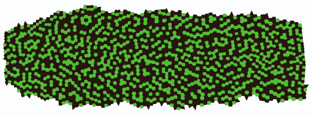 **(0.029)**	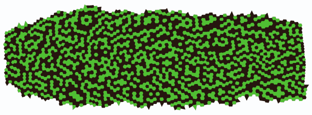 **(0.146)**	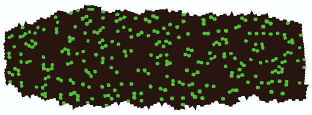 **(0.526)**
	TL2	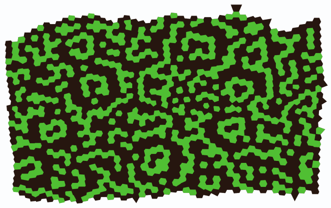 **(0.078)**	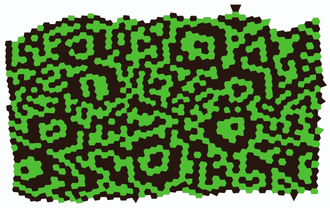 **(0.044)**	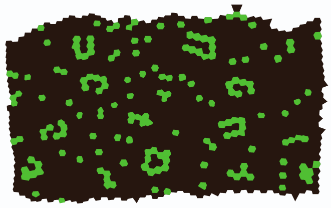 **(0.520)**
	SM1	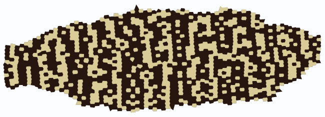 **(0.372)**	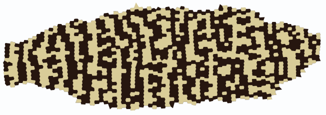 **(0.397)**	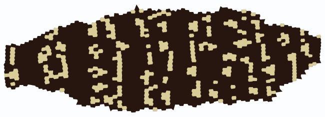 **(0.037)**

Table S4 | Exchanging sets of RD parameters among individuals within and between species (ocellated lizards *vs.* black and white tegu); related to Figures 2 and 3 — Colour patterns of two ocellated lizards (E_{16D}= 0.16 for TL1 *versus* TL2) and one tegu (SM1) simulated after exchanging their optimised RD parameters. Numbers in parentheses indicate E_{16D} errors when compared to the observed adult pattern.
