# Supplementary material for: Modeling convergent scale-by-scale skin color patterning in multiple species of lizards
Source: Curr Biol. 2022 Dec 5;32(23):5069–5082.e13. doi: 10.1016/j.cub.2022.10.044 (PMC9763091; doi:10.1016/j.cub.2022.10.044)
Supplement: Data S1. 16D nearest-neighbor error vectors (ocellated lizard TL1) projected in PC1-PC2-PC3 space, related to Figure 2 — Interactive 3D graph (xhtml format for viewing in web browsers) plotting all error vectors for sCA, Lenz-Ising and RD simulations (cf. main Figure 2A). [file mmc2.zip › Data_S1.xhtml]

Neighbourhood Statistics Errors TL1


# Neighbourhood Statistics Errors - Ocellated lizard TL1 PC1- PC2- PC3
